# Graph neural processes for molecules: an evaluation on docking scores and strategies to improve generalization

**DOI:** 10.1186/s13321-024-00904-2

**Published:** 2024-10-23

**Authors:** Miguel García-Ortegón, Srijit Seal, Carl Rasmussen, Andreas Bender, Sergio Bacallado

**Affiliations:** 1 Statistical Laboratory, University of Cambridge, Wilberforce Rd, Cambridge, CB3 0WA UK; 2 Department of Engineering, University of Cambridge, Trumpington St, Cambridge, CB2 1PZ UK; 3 Department of Chemistry, University of Cambridge, Lensfield Rd, Cambridge, CB2 1EW UK; 4 Imaging Platform, Broad Institute of MIT and Harvard, 415 Main Street, Cambridge, MA 02142 USA

## Abstract

**Abstract:**

Neural processes (NPs) are models for meta-learning which output uncertainty estimates. So far, most studies of NPs have focused on low-dimensional datasets of highly-correlated tasks. While these homogeneous datasets are useful for benchmarking, they may not be representative of realistic transfer learning. In particular, applications in scientific research may prove especially challenging due to the potential novelty of meta-testing tasks. Molecular property prediction is one such research area that is characterized by sparse datasets of many functions on a shared molecular space. In this paper, we study the application of graph NPs to molecular property prediction with DOCKSTRING, a diverse dataset of docking scores. Graph NPs show competitive performance in few-shot learning tasks relative to supervised learning baselines common in chemoinformatics, as well as alternative techniques for transfer learning and meta-learning. In order to increase meta-generalization to divergent test functions, we propose fine-tuning strategies that adapt the parameters of NPs. We find that adaptation can substantially increase NPs' regression performance while maintaining good calibration of uncertainty estimates. Finally, we present a Bayesian optimization experiment which showcases the potential advantages of NPs over Gaussian processes in iterative screening. Overall, our results suggest that NPs on molecular graphs hold great potential for molecular property prediction in the low-data setting.

**Scientific contribution:**

Neural processes are a family of meta-learning algorithms which deal with data scarcity by transferring information across tasks and making probabilistic predictions. We evaluate their performance on regression and optimization molecular tasks using docking scores, finding them to outperform classical single-task and transfer-learning models. We examine the issue of generalization to divergent test tasks, which is a general concern of meta-learning algorithms in science, and propose strategies to alleviate it.

**Supplementary Information:**

The online version contains supplementary material available at 10.1186/s13321-024-00904-2.

## Introduction

A major difficulty in the application of machine learning (ML) to molecular property prediction in drug discovery is the scarcity of labeled data. Experimental assays are expensive and time-consuming, and data collection is biased towards certain bioactivities (e.g. protein targets deemed medically relevant or commercially profitable) or molecules (e.g. those that are easier to acquire or synthesize). As a result, chemoinformatic datasets are highly sparse and non-overlapping. In a typical pharmaceutical company’s chemical library, it is estimated that less than 1% of all the compound-assay pairs have been measured [1]. Even more strikingly, public databases are as little as 0.05% complete [1, 2].

Meta-learning, or “learning to learn”, is a machine-learning paradigm that attempts to achieve fast adaptation to novel tasks given a small number of labeled datapoints [3]. The meta-learning setting is similar to transfer learning in that it attempts to take advantage of existing information to improve predictions on downstream tasks. However, instead of transferring knowledge from a single pre-training task with many labels, meta-learning attempts to transfer knowledge from multiple meta-training tasks with few labels each. Later, during meta-testing, the model is evaluated on unseen tasks, using a few labeled datapoints from each task as examples. These example points encode information about the meta-test task and are called the contexts. In turn, the query points of interest that we want to predict for each meta-task are called the targets. The ability to learn from meta-training tasks without overfitting and generalizing to novel meta-testing tasks is called meta-generalization.

The meta-learning setting may be appropriate in molecular property prediction [4], since measurements from many different molecular tasks have been collected historically and could be used for meta-training. Examples of molecular tasks are physicochemical properties, protein binding affinities, phenotypic assays or ADMET endpoints [5, 6]. Typically, each task comprises too few datapoints to train a large neural model, but collectively a large set of bioactivities may be useful to learn biases of molecular functions, as well as molecular representations. However, given the sheer diversity of molecular tasks that are available, extra care should be taken to ensure that the biases learnt during meta-training are adequate for meta-testing. For example, tasks related to physicochemical properties, which are intrinsic to molecules, may be very different from cell assays, which depend on the complex interplay between molecules and a biological system [7].

In addition to data efficiency and learning from sparse datasets, another feature that is desirable is the ability to produce uncertainty estimates [8]. Well-calibrated uncertainty estimates are helpful in settings that involve molecular selection and subsequent experimental validation, such as Bayesian optimization (BO) or virtual screening (VS), since they allow users to balance exploration and exploitation in a principled manner. For example, the selected set could combine some novel but uncertain molecules with others more conservative but certain. Such strategies could help prevent committing to the wrong set of molecules early in development, which is extremely costly [9]. Neural processes (NPs) [10, 11] are a family of models for probabilistic meta-learning that can estimate the uncertainty of each prediction.

Meta-learning for molecular property prediction is a relatively new but rapidly growing area in ML research. Nguyen et al. [4] was an early study that showed the benefits of model-agnostic meta-learning (MAML) for bioactivity classification. However, MAML suffers from lack of robustness during meta-training [12], and the model used in this study did not provide uncertainty estimates, which reduces interpretability and prevents BO for molecular optimization. More recently, Chen et al. [13] presented ADKF-IFT, a deep kernel Gaussian process (GP) for molecular meta-learning. Like NPs, deep kernel GPs are neural models that produce uncertainty estimates. However, the ADKF-IFT method was benchmarked on FS-Mol [14], which is susceptible to overfitting due to random splitting, and it is comparatively difficult to implement, which prevents re-training on novel datasets by chemoinformatics practitioners. NPs for molecules were recently explored for the first time [15–17], yielding promising results. However, the benchmarking experiments in these early studies were limited, not assessing uncertainty calibration, applicability domain or the effect of choosing fingerprint (FP) or molecular graph (MG) representations. In general, hand-engineered representations like FP are considered advantageous in single-task, low-data settings, whereas data-driven representations derived from MGs may provide benefits in single-task, high-data settings. However, it is unclear whether FPs or MGs should be preferred in the multi-task, low-data setting of meta-learning. The NP model by Chan et al. [17] addressed function heterogeneity in bioactivity datasets by clustering similar assays, effectively denoising predictions from across-assay variability. This strategy could be complementary to ours, which is based on parameter adaptation.

In chemoinformatics research, transfer learning in the low-data setting has been previously approached from the perspective of imputation of sparse datasets [18, 19]. Alchemite [1] is a commercial model for imputation of bioactivities that resembles NPs in that information about the test task is encoded implicitly in the context points. In principle, this makes NPs and Alchemite applicable to any molecular task as long as some molecules have been measured and can be used as context points. In contrast, a related family of models that rely on explicit protein representations are proteochemometric models, which predict affinity values from protein-ligand pairs [20, 21]. Proteochemometric models are less general since they can only be applied to tasks associated with a single known protein, since they depend on explicit protein representations.

In this work, we study the application of NPs to molecular properties and compare their performance to single-task, transfer-learning and meta-learning baselines that are popular in chemoinformatics or in molecular ML, using either a FP or MG representation. We conduct our evaluation on DOCKSTRING, a dataset of docking scores and benchmarking framework for regression and molecular optimization that includes labels for 260k molecules and 58 molecular tasks corresponding to 58 protein targets from diverse protein families [22]. DOCKSTRING provides a controlled environment where each molecule is annotated for every task, and where task similarity can be objectively quantified as the correlation between docking scores of protein targets. These qualities make it ideal for benchmarking molecular meta-learning, since it becomes possible to test few-shot learning (FSL) across a range of dataset sizes, and to assess meta-generalization to novel tasks across a range of similarity to training tasks. We observe that NPs on molecular graphs outperform other models in FSL experiments, and provide well-calibrated uncertainty estimates that may inform the reliability of their predictions. We study the applicability domain of NPs and observe that their performance in distant regions of chemical space remains higher than that of other models. In addition, we suggest a simple yet effective fine-tuning strategy that improves meta-generalization. Finally, we perform a Bayesian optimization (BO) experiment that showcases the potential of NPs for finding optimal molecular candidates within a chemical library.

## The challenge of meta-generalizing to real-world tasks

One potential obstacle to using meta-learning for molecular property prediction, especially for bioactivity prediction, is the heterogeneity of molecular tasks [17, 23]. Meta-learning attempts to transfer knowledge from a set of tasks with already existing data (the meta-training tasks) to another set of novel tasks with little to no existing data (the meta-test tasks). Crucially, the meta-training tasks must be similar enough to the meta-test tasks, and the heterogeneity among meta-training tasks must be of similar magnitude to the heterogeneity between meta-train and meta-test tasks, for the knowledge transfer to help predict meta-test tasks. However, many meta-learning benchmarks in the ML literature are limited to tasks of low diversity, which may inflate the perceived robustness of meta-learning methods. For example, MAML for regression was evaluated on a simple dataset of 1D sinusoid functions of the same frequency [3], and NPs were evaluated on the MNIST dataset of handwritten digits [10, 11], splitting images randomly into training and test sets even though images in the same digit class (0 to 9) are clearly highly homogeneous.

**Fig. 1:**
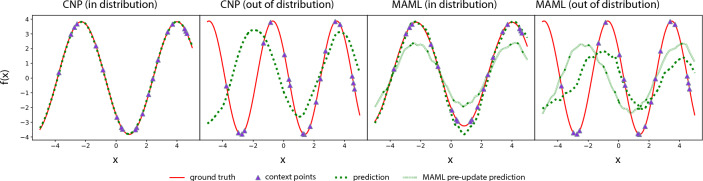
Meta-generalization experiment on 1D sinusoids. CNPs (left) and MAML (right) meta-trained on sinusoids with frequency $\rho =1$  generalize to similar test functions. However, changing the frequency to $\rho = 1.5$  leads to catastrophic loss of generalization. Quantitative results are shown in Table F.1. This figure is inspired by Finn et al. [3]

For an empirical illustration of the challenge of meta-generalization even in simple functions, we consider the set of 1D sinusoids used to evaluate MAML regression [3]. In this benchmark, a task is defined by predicting the value of a function of the form $A\sin (\rho (x-B))$ at different values of the input *x*. Different tasks have different values of *A* and *B*, but the same unit frequency $\rho =1$. We performed a simple experiment to show that even a slight modification to the meta-test tasks, such as a small change in sinusoid frequency, is enough to compromise meta-generalization in both MAML and conditional NPs (CNPs) (Fig. 1 and Supplementary Section F for details). If a small change in a simple function class over a 1D input space can disrupt meta-generalization, mismatches between meta-training and meta-testing in the real world, where one may encounter highly heterogeneous functions over high-dimensional input spaces, are likely to cause problems too.

Scientific applications such as bioactivity prediction in preclinical studies may require meta-generalizing to divergent meta-test tasks, since mismatches between meta-training and meta-testing may be unavoidable in a research context. First, by definition research involves an attempt to acquire new knowledge, which means that novel tasks of interest (e.g. novel protein targets, novel cell assays, etc) may not have any known close relatives available for meta-training. Second, if the overlap of labeled datapoints among tasks is limited due to sparsity, it may be difficult to identify the closest task relatives available, which in turn may make it difficult to build an optimal meta-training set. Finally, public bioactivity databases integrate data from a large variety of sources and assays, which leads to extreme task heterogeneity even among seemingly comparable tasks [17, 23]. This heterogeneity was recently quantified by Landrum and Riniker, who developed a “maximal curation” strategy to try to identify affinity assays in ChEMBL that were equivalent, with a view to combining them [23]. In addition to checking that assays were carried out against the exact same protein target and variant (e.g. splice variant, mutant, etc), they also ensured that assays employed the same technology (as per the BioAssay Ontology [24]) and that proteins were expressed in the same strain and purified from the same subcellular fraction. Even after such curation, 48% of overlapping measurements differed by more than the median experimental error. This result points at high heterogeneity even within seemingly equivalent assays. Interestingly, this high variability was observed not only in IC50 measurements, but also in $K_i$, which is traditionally believed to be more robust [23]. Similar results have been independently reported by others: Chan et al found that out of 190 pairs of ChEMBL IC50 assays against the same protein, only 34 (17.9%) had a Pearson correlation greater than 0.7 [17]. In light of this heterogeneity, meta-learning methods which fail to meta-generalize to divergent test tasks may be of limited practical value to bioactivity prediction, a challenge that current meta-learning benchmarks may overlook.

While these difficulties complicate the application of meta-learning to molecular property prediction, transferring knowledge across very different, even seemingly unrelated tasks has been previously achieved through parameter adaptation [25]. Specifically for molecules, recent work by Formont et al. [26] has shown that fine-tuning may be more robust to shifts in the distributions of molecular labels than traditional meta-learning. The authors noted that this feature increases their practical utility and is currently overlooked in the literature [26]. In this paper, we explore a fine-tuning strategy to adapt the parameters of NPs to novel tasks, as a way to improve generalization during meta-testing. As a benchmarking task, we focus on the prediction of docking scores from the DOCKSTRING dataset, where tasks are different protein targets [22]. This provides us with a controlled environment where the divergence between tasks can be quantified exactly, which enables studying meta-generalization across a range of divergences.

## Methods

### Neural processes (NPs)

Consider a meta-training dataset with observations of real-valued functions or tasks $f_1, \ldots , f_n$, $f_i : {\mathcal {X}} \rightarrow {\mathbb {R}}$ (we use the terms *function* and *task* interchangeably in this section, which are not to be confused with the notion of *targets* described below). In this paper, ${\mathcal {X}}$ represents the space of chemically feasible molecules, and $x \in {\mathcal {X}}$ refers to a single molecule represented either as a fingerprint vector (FP) or as a molecular graph (MG). Each molecular function $f_i$ is observed at a set of $O_i$ input points $x_o^i \in {\mathcal {X}}^{O_i}$, with known labels $y_o^i = \left( y_{o,1}^i, \ldots , y_{o,O_i}^i\right)$, where $y_{o,j}^i = f_i(x_{o,j}^i)$. Additionally, consider a *meta-test* function *f*, observed at a small set of *C* *context* points $\left( x_c, y_c\right) = \left( \left( x_{c,1}, y_{c,1}\right) , \ldots , \left( x_{c,C}, y_{c,C}\right) \right)$. Our goal is to predict the values $y_t$ of *f* at a set of *T* *target* locations $x_t \in {\mathcal {X}}^T$ as accurately and efficiently as possible, using the example context points $\left( x_c, y_c\right)$ and observations from the example functions $f_i, \ldots , f_n$ or *meta-training data*.

A neural process (NP) is a model for meta-learning that aims to describe the epistemic or Bayesian predictive distribution of the target outputs given the target inputs, context data, and meta-training data. This predictive distribution will be denoted $q\left( y_t \mid x_c, y_c \, ; x_t\right)$, suppressing the dependence on meta-training data. We use semicolon notation to differentiate contexts and targets, e.g., $q\left( y_t \mid x_c, y_c \, ; x_t\right)$ in a predictive density or ${\mathcal {L}}(y_t \mid x_c, y_c\, ; x_t)$ in an objective function. This distinction will be helpful in later sections, where the context outputs could themselves be predicted, e.g., $q\left( y_c \mid x_c, y_c \, ; x_c\right)$ or ${\mathcal {L}}(y_c \mid x_c, y_c \,; x_c)$.

NPs assume conditional independence between target points and a Gaussian predictive distribution:

$$\begin{aligned} q\left( y_t \mid x_c, y_c\, ; x_t\right) = \prod _{j=1}^{T} {\mathcal {N}}\left( y_{t,j} \, ; \, \mu _\theta \left( x_{t, j},x_c,y_c\right) , \sigma ^2_\theta \left( x_{t, j},x_c,y_c\right) \right) , \end{aligned}$$

where the mean and variance, $\mu _\theta$ and $\sigma ^2_\theta$, are neural functions whose parameters $\theta$ will be fit using the meta-training data. For any input $x\in {\mathcal {X}}$, the functions $\mu _\theta (x,x_c,y_c)$ and $\sigma _\theta ^2(x,x_c,y_c)$ are computed in three steps. First, each context point $\left( x_{c,j}, y_{c,j}\right)$ is mapped by an encoder network $h_\theta$ to a local datapoint representation $r_j$. Then, all context encodings $r_j$ are combined into a global function encoding *r* through a commutative operation, usually the sum or the mean. Commutativity guarantees invariance to contexts’ permutations. Finally, a decoder network $g_\theta$ maps the function encoding *r* and the input location *x* to the predictive mean and variance, respectively.

In this paper we use two flavors of NPs: the conditional NP (CNP) [10] and the latent NP (LNP) [11]. In the CNP, the decoding step is deterministic. The latent LNP is slightly more complex; decoding involves sampling a latent random variable *z* from an approximate posterior $\tilde{q}_\phi$, which is then fed as an input instead of *r* to the decoder network $g_\theta$. A more precise description of the encoding and decoding process is provided in Supplementary Section A.1.

The parameters of the CNP $\psi =\{\theta \}$ are trained by backpropagation from the predictive log-likelihood ${\mathcal {L}}_\psi (y_t \mid x_c, y_c \,; x_t)=\log q_\theta (y_t \mid x_c, y_c\, ; x_t)$. During meta-training, each meta-train function $f_i$ is seen once every epoch, but not all observations $\left( x_o^i, y_o^i \right)$ are used at each iteration. Rather, the $O_i$ observations are randomly subsampled to create two disjoint sets: a context set $\left( x_c^i, y_c^i\right)$ and a target set $\left( x_t^i, y_t^i\right)$, with sizes $C_i$ and $T_i$ respectively, $C_i + T_i \le O_i$. The predictive log-likelihood on the current targets is optimized given the current contexts. Therefore, the final objective is

1 $$\begin{aligned} {\mathbb {E}}\Big [ \frac{1}{n} \sum _{i=1}^{n} {\mathcal {L}}_\psi \left( y_t^i \mid x_c^i, y_c^i\, ; x_t^i\right) \Big ], \end{aligned}$$

where the expectation is with respect to the random sampling procedure. $C_i$ and $T_i$ can themselves be stochastic: in our experiments, we sample them uniformly from [20, 150) at each iteration. We find that this randomization is key to uncertainty quantification, making the model robust to varying context and target sizes at test time. In section Randomization of context and target sets protects NPs from overfitting we investigate the influence of these hyperparameters on the generalization of NPs.

The parameters of the LNP $\psi =\{\theta ,\phi \}$ are trained by backpropagation from a function ${\mathcal {L}}_\psi (y_t \mid x_c, y_c \,; x_t)=\log q_\theta (y_t \mid x_c, y_c\, ; x_t) + \rho (\phi ,x_o,y_o)$, where a regularization term $\rho (\phi ,x_o,y_o)$ reduces the sensitivity of the encoder to any given sample (Supplementary Section A.2). This regularization is motivated by a variational Bayesian argument [11]. Meta-training is performed by randomising the context and target sets, as outlined previously, yielding an objective of the form (1).

### Computational considerations of NPs

A forward pass of *N* datapoints through a neural network (NN) with fixed architecture has runtime ${\mathcal {O}}(N)$. A forward pass of *T* target points through a NP with fixed architecture, conditioned on *C* context points, requires evaluating the encoder $h_\theta$ on the contexts and the decoder $g_\theta$ on the targets, which results in runtime ${\mathcal {O}}(C + T)$. If the NP is evaluated on ${\mathcal {T}}$ tasks simultaneously, the complexity becomes ${\mathcal {O}}\big ({\mathcal {T}}(C + T)\big )$.

In practice, forward propagation of NNs and NPs is parallelized so that a whole batch is processed at the same time, with the size of the batch *B* chosen so as to fit the memory of the GPU. A batch for a NN will contain $B_N$ of the *N* datapoints. Assuming each datapoint is a vector of length *M*, a NN batch will have dimensions $B_N \times M$ (2D). In contrast, a batch for a NP will contain $B_{\mathcal {T}}$ of the ${\mathcal {T}}$ tasks, so it will have dimensions $B_{\mathcal {T}} \times (C + T) \times M$ (3D). Higher-dimensional datapoints (e.g. 2D adjancency matrices if representing molecules as graphs) will result in even higher-dimensional batches.

The higher dimension of NP batches with respect to NN batches may result in high memory usage, which may make it difficult to fit the GPU. In practice, running out of memory can be avoided by choosing a lower batch size $B_{\mathcal {T}}$ or by implementing automated checks in the NP encoder $h_\theta$ and decoder $g_\theta$ so that, if the context size *C* or the target size *T* in a batch are high, contexts and targets in that batch are chunked and processed in several passes rather than in a single pass.

### Effective epochs

Since only some observations of a function $f_i$ are sampled as contexts or targets every epoch, how often an individual observation is seen will depend on the sample size relative to the total number of observations for that function $O_i$, which for molecular datasets can vary widely (Supplementary Section B). In order to homogenize training across varying sample sizes and observed sets, we introduce the concept of *effective epochs* $e_e$, which we define as the average number of times an observed datapoint is seen during training. This quantity is calculated as

$$\begin{aligned} e_e^i = e \; \frac{{\bar{n}}}{O_i}, \end{aligned}$$

where *e* is the number of epochs and ${\bar{n}}$ is the average sample size (sample size could itself be random). In our experiments, the average number of contexts and targets while training is the same in every experiment, so effective epochs refer to views of an observation both as context and target.

### Parameter adaptation during meta-testing

When a NP is applied to a test function *f* with contexts $\left( x_c, y_c \right)$, its predictions on the contexts themselves may be inaccurate. In particular, if a NP fails to meta-generalize, the predicted context density

$$\begin{aligned} q(y_c \mid x_c, y_c \,; x_c) = \prod _j^C {\mathcal {N}}(y_{c,j}\, ; \mu _\theta (x_{c,j},x_c,y_c), \sigma _\theta ^2(x_{c,j},x_c,y_c)) \end{aligned}$$

may be inadequately low, even though the contexts are given as input. In this situation, the loss on the context predictions ${\mathcal {L}}_\psi (y_c \mid x_c, y_c \,; x_c)$ can be exploited to adapt the weights to the test function *f*, potentially improving meta-generalization. We studied two strategies for parameter adaptation by backpropagation during meta-testing: fine-tuning and a single step of gradient descent on a NP trained with model-agnostic meta-learning (MAML) [3]. MAML is a meta-learning training regime that involves nested gradient descent and is notorious for displaying instability during training [12]. Although we implemented improvements from the successor MAML++ [12] to increase robustness, we still observed high variance in MAML-trained models, and average performance was lower than that of fine-tuning. Therefore, for the remainder of this work we will focus on fine-tuning. See Supplementary Section C for a description of MAML and to view MAML results.

We fine-tuned NPs by mimicking the meta-training procedure on the test function *f* (Algorithm 1). To this end, at each epoch we split the test function’s contexts $\left( x_c, y_c \right)$ into new contexts and new targets, as if they were the observations of a meta-train function. Then, we used the new contexts $\left( x_{c'}, y_{c'} \right)$ and targets $\left( x_{t'}, y_{t'}\right)$ to evaluate the NP’s objective ${\mathcal {L}}_\psi (y_{t'} \mid x_{c'}, y_{c'} \,; x_{t'})$ and backpropagate as usual. In this way, fine-tuning during meta-testing resembled meta-training, but instead of iterating over many train functions every epoch, it focused on a single test function. As in meta-training, the new contexts and targets were disjoint, with sizes $C' + T' \le C$. We set the number of desired effective epochs $e_e$ as a hyperparameter, and calculated the required number of actual epochs *e* required to achieve the desired $e_e$ based on the original context size *C* and the number of new targets $T'$.

In our experiments, we adapted the weights for 20 effective epochs, sampling $C' = T' = 20$ new contexts and targets every iteration. The only exception from this protocol was the few-shot learning (FSL) experiment with 20 observations, where we used $C' = T' = 5$. To minimize the risk of overfitting, we fine-tuned the last layers and froze the rest. Specifically, we always adapted the last two layers of the decoder network $g_\theta$, and in LNPs we also adapted the last two layers of the encoder $g_\phi$ (see Appendices A.1 and E for architectural notation and details). The latter allowed us to optimize the regularization term of the LNP objective ${\mathcal {L}}_{\theta , \phi }$, which depends on the encoder (Supplementary Section A.2).

**Algorithm 1:**
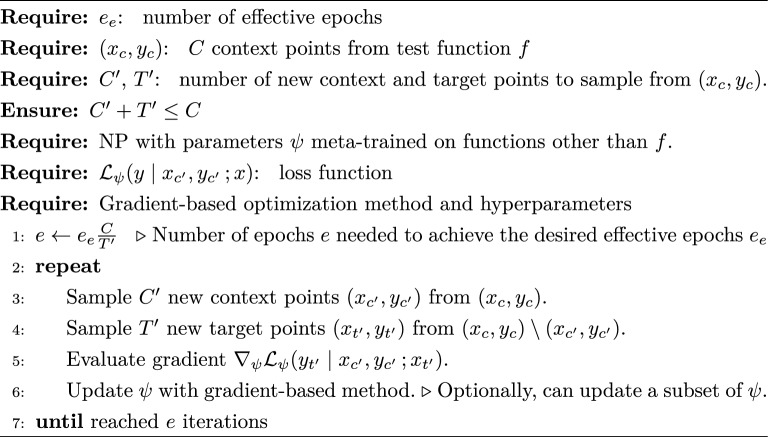
Fine-tuning: Adapting NP parameters to test function *f*

### Molecular graph neural processes (MG-NPs)

In order to apply NPs to molecular graphs (MGs), we expanded the encoder module with a graph neural network (GNN) that processed atom and bond features and implemented message passing with attention. Our architecture was inspired by the Attentive FP model [27], with the differences that we processed atom and bond features with a small network before and after message passing, we removed gated recurrent units (GRUs) [28] and we changed the attention mechanism to query-key-value (QKV) [29]. These changes were aimed at speeding computation while maintaining expressivity.

Prior to message-passing, atoms and bonds were processed with a small fully-connected neural network (FNN, 2 layers and 50 hidden neurons) to encode atomic features into vectors of length 25 and bond features for each atom *i* into matrices of size $n_i \times 25$, where $n_i$ is the number of neigbours of atom *i*. Then, bond feature matrices were summed along the row dimension to yield a single bond vector of length 25 for each atom. This was concatenated to the previous atomic encoding, such that the final atomic encoding (before message passing) was a vector of length (50). Then, we updated the atomic encodings with 3 iterations of message passing from direct atomic neighbours, calculating attention coefficients with a QKV mechanism. The queries and keys are computed with a single-layer FNN. After the initial 3 iterations of message passing, a second stage began where an imaginary “superatom” connected to all atoms was added to the graph, and another 3 iterations of message-passing were performed. The encoding of the superatom is treated as the representation for the whole molecule. As a final step, the superatom encoding was passed through a small FNN to yield the final molecular representation, also of length 50.

Implementation details of the MG-NP and all other baseline models are provided in Supplementary Section E.

### Dataset and data splits

DOCKSTRING is a bundle for benchmarking ML models for molecules using docking scores as objective properties [22]. It consists of a dataset of scores and poses and a set of evaluation tasks that reflect the model’s performance in regression, virtual screening and multiobjective molecular optimization. The dataset includes Autodock Vina scores for every ligand-target pair in 260k molecules from ExCAPE [30] and 58 targets from DUD-E [31]. While other popular benchmark datasets in ML for drug discovery contain sparse annotations, in the sense that not every molecule included is labeled for every property considered (e.g. MoleculeNet [32], FS-Mol [14]), the DOCKSTRING ligands have docking scores for every target, which makes DOCKSTRING suitable to the design of new benchmark tasks in multi-task learning, transfer learning or meta-learning. Note that docking scores in DOCKSTRING are not intended for downstream applications, but rather as proxy properties for benchmarking that are loosely reflective of the 3D interactions between a molecule and a protein structure. Thus, the purpose of DOCKSTRING scores is not to perfectly reflect binding affinity or to improve docking algorithms, but to enable a reproducible and fair comparison of ML models on the same set of benchmark tasks. To this end, ligand preparation and docking are performed with fixed random seeds so as to maximize repeatability across different runs, and each benchmark task has well-defined training and test sets to ensure a fair model comparison. In the DOCKSTRING regression benchmark, roughly 220k compounds are assigned to the training set and 40k are assigned to the test set. They are split by chemical scaffolds to reduce the risk of data leakage from chemical analogues [33–35]. The regression benchmark focuses on 5 diverse proteins, some of which have scores that are relatively easy to predict, and some which are harder, so as to reflect a range of prediction difficulty.

**Fig. 2:**
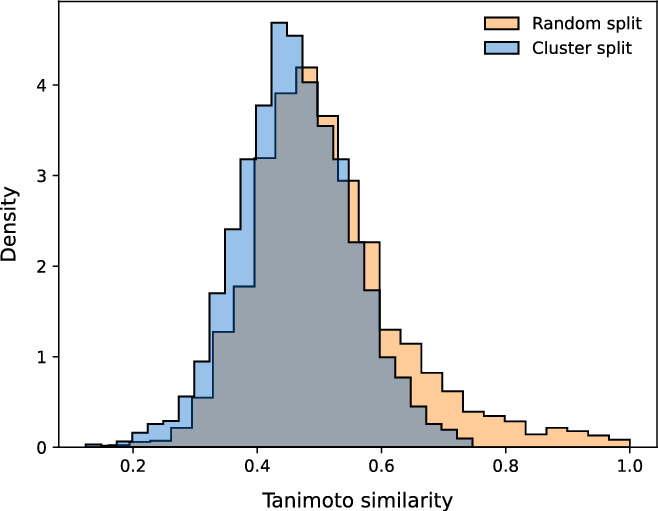
Distribution of the Tanimoto similarity of every molecule in the test set to its closest neighbour in the training set, either in the DOCKSTRING split (*dtrain* and *dtest*) or in an example random split. Tanimoto similarity values were computed on Morgan fingerprints of length 1024 and radius 3, calculated with RDKit

**Fig. 3:**
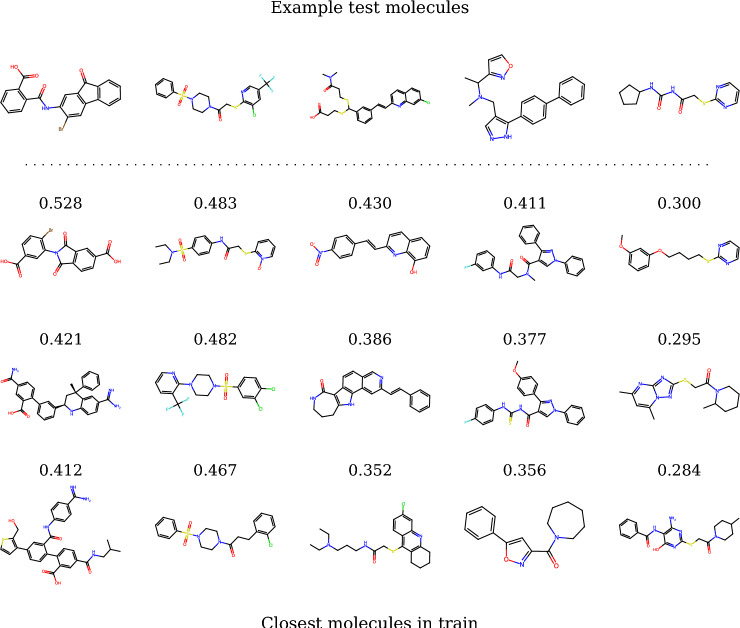
5 random molecules from the test set and their three closest neighbours in the training set. Tanimoto similarity values were computed on Morgan fingerprints of length 1024 and radius 3, calculated with RDKit

*Few-shot learning (FSL)* For our meta-learning evaluation, we needed to create splits not only across datapoints (*dtrain* and *dtest*) but also across tasks or functions (*ftrain* for meta-train and *ftest* for meta-test). Regarding datapoints, in the few-shot learning (FSL) experiments, we respected the scaffold splits of the DOCKSTRING regression benchmark, sampling 2.5k molecules from the training set and 2.5k molecules from the test set for our *dtrain* and *dtest* respectively. To confirm that this split was more stringent than a random one, we visualized (Fig. 2) the distribution of the Tanimoto similarity of every molecule in *dtest* to its closest neighbour in *dtrain* (blue histogram), and compared it to the homologous distribution arising from a random split (orange histogram). As expected, the random split led to chemical analogues spread across the training and test set, as reflected by the heavy tail of high Tanimoto similarities. In contrast, the DOCKSTRING split was more strict and prevented such analogues between *dtrain* and *dtest*. We also visualized a random sample of datapoints from *dtest* and their closest three molecules in *dtrain* according to Tanimoto similarity (Fig. 3). We found that the similarity values of the closest neighbours were relatively low, and no neighbours shared the same scaffold across training and test sets.

**Fig. 4:**
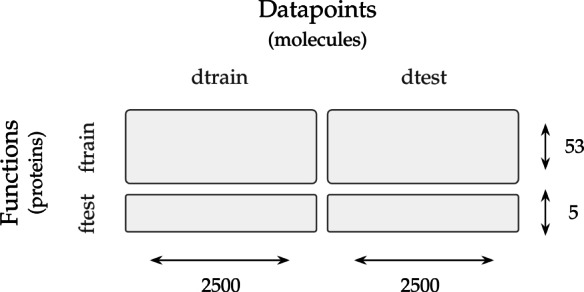
Data split in the FSL experiments. Meta-learning models were trained on *ftrain*, *dtrain* and tested on *ftest*, *dtest*, using points from *ftest*, *dtrain* as contexts

**Fig. 5:**
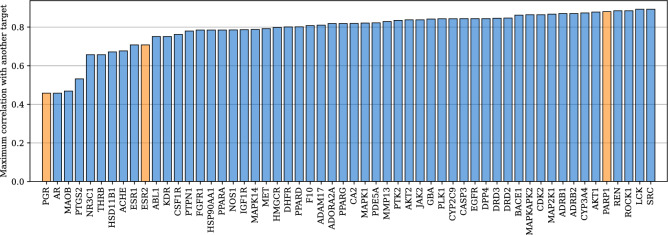
Maximum Pearson correlation of the docking scores of each protein in DOCKSTRING to those of any other protein. The maximum correlation of each protein suggests how challenging it would be for meta-generalization. We identified three proteins from the DOCKSTRING regression benchmark (orange bars) that represented a range of dissimilarity to other proteins: PARP1 (highly correlated, very similar), ESR2 (medium correlation and similarity) and PGR (low correlation and similarity). We selected these proteins as our meta-test set, and all proteins not in the regression benchmark as meta-training set. In this way we aimed to illustrate meta-generalization across a range of correlations

Regarding functions,we selected three proteins from the regression benchmark (PARP1, ESR2 and PGR) as meta-test *ftest* and kept the 53 training proteins from the benchmark as meta-train *ftrain* (Fig. 4). The three proteins chosen for meta-testing reflected a range of similarity (high, medium and low) to other proteins, as indicated by the Pearson correlation of their docking scores on the full dataset of 260k molecules (Fig. 5). This allowed us to examine the generalization of transfer learning and meta-learning methods to novel tasks which were either close or distant to previously seen tasks.

The protein and datapoint splits of the FSL experiment were used by each model family as follows:

- Meta-learning models were meta-trained on *ftrain*, *dtrain*. During meta-testing, they used random points from *ftest*, *dtrain* as contexts and all points from *ftest*, *dtest* as targets. Metrics were calculated on all points from *ftest*, *dtest*.
- Transfer-learning models were pre-trained on *ftrain*, *dtrain* and fine-tuned on random points from from *ftest*, *dtrain*. Metrics were calculated on all points from *ftest*, *dtest*.
- Single-task models were trained on points randomly sampled from *ftest*, *dtrain*. Metrics were calculated on all points from *ftest*, *dtest*.

As an alternative for benchmarking FSL other than DOCKSTRING, we also considered using FS-Mol [14], a dataset for molecular meta-learning that includes bioactivity values from ChEMBL. However, FS-Mol only considers classification tasks, whereas NPs are usually used for regression; its labels are non-ovelapping across molecules, which precludes flexible subsampling; and it splits molecules randomly, which may lead to data leakage and an overestimation of performance [33–35]. In contrast, DOCKSTRING is a complete matrix with full overlap of annotations across tasks, which allows flexible sampling of variably-sized subsets, and it splits molecules by scaffold, which minimizes the risk of data leakage from chemical analogues (Figs. 2 and 3).

*Bayesian optimization (BO)* We created a library of 60k compounds for the BO experiments by sampling 30k molecules from the DOCKSTRING regression training set and 30k from the test set. As objective functions to optimize, we used the druglike F2 and the selective JAK2 objectives from the DOCKSTRING optimization benchmark (Supplementary Section H). These objective functions were treated as meta-test tasks by the meta-learning models: at each iteration of BO, all molecules seen up to that point were used as contexts and all 60k molecules in the library were used as targets (at each iteration, we selected the best predicted molecules from these targets which had not been seen yet). For meta-training tasks, we created an augmented dataset where scores were transformed either linearly (scalar multiplication and linear combination) or non-linearly (by taking the minimum or the maximum between a given score and the median of the score distribution for the corresponding protein target), and were combined with the quantitative estimate of drug-likeness (QED). We created 869 such transformed tasks; this odd number arose from creating an augmented dataset of 1000 transformations and discarding those which included F2, JAK2 or LCK, since those three proteins were part of the DOCKSTRING objective functions. We meta-trained meta-learning models on the 869 augmented functions. We trained two different MG-CNPs: one on the whole 60k molecules of the BO library and one on the 2.5k *dtrain* molecules from the FSL split.

### Molecular representations

All models used either a fingerprint (FP) or molecular graph (MG) representation. We computed binary Morgan FPs [36] of length 1024 and radius 3 with RDKit [37]. MGs were obtained from SMILES strings [38], using RDKit to extract the connectivity graph and the atom and bond features. Connectivities were represented in PyTorch as adjacency matrices. Atom features consisted of one-hot vectors indicating the atom type, the number neighbouring hydrogens, the number of neighbouring heavy atoms, the formal charge, the hybridisation type, whether the atom was placed within a ring, whether the atom was in an aromatic region and whether the atom was chiral. Atom features also included, as real numbers, the atomic mass, the Van der Waals radius and the covalent radius. Bond features included one-hot vectors with the bond type (single, double, tripe or aromatic), whether the bond was conjugated, whether it was part of a ring, and the stereoisometry type, if any.

All models labeled with “FP” used the fingerprint representation and all models labeled with “MG” used the molecular graph representation. GNNs also used the MG representation. The ADKF-IFT baseline model came pre-trained and used its own FPs (Morgan FPs of length 2048 and radius 2). The dummy regressor simply predicted the mean of the training molecules and did not use any representation.

## Results

### Few-shot learning (FSL)

We evaluated few-shot learning by MG-NPs on docking scores from the DOCKSTRING dataset and compared their performance to a variety of baselines, including single-task, transfer learning and meta-learning models. Few-shot learning (FSL) is a ML paradigm where models are trained using just a handful of datapoints, typically between 5 and 50. In the meta-learning setting, FSL is achieved by first meta-training the model on several tasks, each of them with a low-to-medium number of labels, and then adapting the predictions of the model to a novel meta-test task for which just a few datapoints are available. In the context of molecular property prediction, the goal of FSL is to predict a new molecular task for which just a few molecules are annotated.

To recreate a low-data setting, we created a small training set by sampling a subset of 2.5k molecules from the DOCKSTRING training set, and a test set by sampling the same number from the test set, which we call the *dtrain* and *dtest* respectively (section Dataset and data splits). We trained all models on the 2.5k molecules from the training set. Single-task models were trained on *ftest, dtrain* and meta-learning models were meta-trained on *ftrain, dtrain*. At test time, we evaluated all models on *ftest, dtest*, with meta-learning models using context points sampled randomly from *ftest, dtrain* (section Dataset and data splits and Supplementary Section E). We inspected a range of numbers of context points, from 20 to 1000. In this way, we aimed to reflect the information available in bioactivity datasets, where the number of observations per task may fluctuate considerably (Supplementary Section B). The range of contexts also allowed us to examine the robustness of NPs, and assess whether their uncertainty estimates were well calibrated over a number of observations.

Table 1 shows the coefficient of determination ($R^2$) for the prediction of the three meta-test proteins: PARP1, ESR2 and PGR. These proteins represent a range of similarity to the meta-training tasks, from high to low, as shown by the correlation of their docking scores to other proteins (Fig. 5). The lower the similarity of a meta-task to meta-training tasks, the more challenging it will be for meta-learning models, and the greater the potential benefit of parameter adaptation. When the mismatch between meta-training and meta-testing was low (as for PARP1), all meta-learning methods outperformed single-task models in the low-data regime, as expected. However, when the mismatch grew (ESR2 and PGR), the biases learnt during meta-training could become detrimental to prediction, leading to poor performance by meta-learning methods. Parameter adaptation by fine-tuning could greatly improve predictions, especially when there was a mismatch. We also attempted parameter adaptation by MAML but it suffered from training instability, yielding large error bars (Table D.1). In general, CNPs performed better than LNPs, and NPs using molecular graphs (MGs) as representations performed significantly better than NPs using fingerprints (FPs). The MG-CNP ranked consistently as the best model in terms of the coefficient of determination $R^2$ by a large margin. However, ADKF-IFT [13], a GP-based model that meta-learns a deep kernel, showed the best calibration of uncertainty estimates according to the negative log predictive likelihood (NLPD, Table D.2).

Our results show that meta-learning MG-NPs can be highly beneficial for molecular property prediction in the low-data setting. We selected the MG-CNP as the best-performing model for more detailed analysis in the following sections.

**Table 1:**
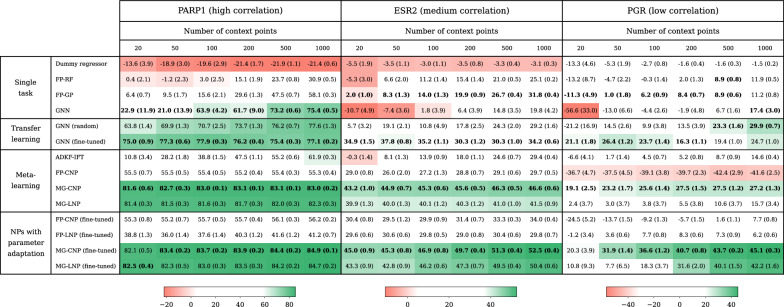
Coefficient of determination ($R^2$) of molecular NP models and baselines in FSL prediction of PARP1, ESR2 and PGR

Each cell shows the mean and standard error (in parentheses) of 10 random repetitions. The MG-CNP ranked consistently as the best model in terms of $R^2$. Fine-tuning led to substantial improvements, especially when the meta-test task was poorly correlated with meta-training tasks (as in ESR2 and PGR) and there were many observations available

### Applicability domain

**Fig. 6:**
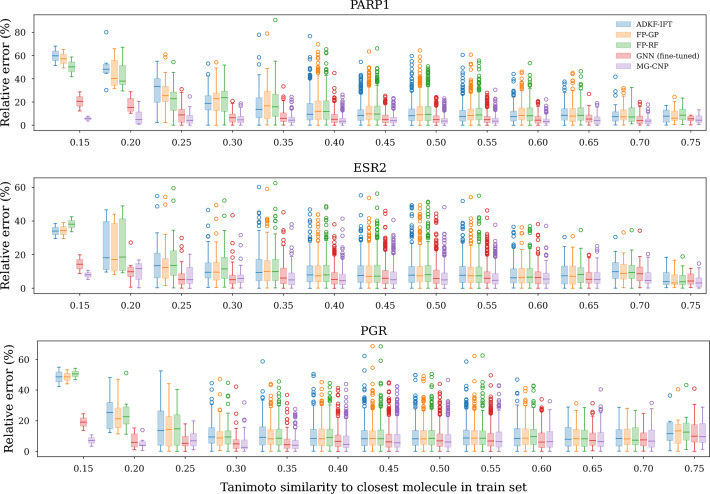
Applicability domain of models within our FSL dataset. We aimed to examine the error of molecules in the test set across a range of distances to molecules in the training set. To this end, we ordered all test molecules by their Tanimoto distance to their closest neighbour in the training set, and assigned each molecule to one of 20 buckets, each of them covering a 0.05-long interval in the [0, 1] range of Tanimoto distances. Then, we computed the relative error for each molecule’s prediction. Relative error $RE_i$ of molecule *i* was defined as $RE_i = \left| \frac{y_i - {\hat{y}}_i}{y_i}\right| \cdot 100$, where $y_i$ represents the true value and ${\hat{y}}_i$ the predicted one. For probabilistic models like GPs and NPs, ${\hat{y}}_i$ was taken to be the predicted mean $\mu _i$. Finally, we plotted the distribution of relative errors of each model within each bucket. The MG-CNP displayed the best performance across all distances within our dataset, and it was closely followed by the fine-tuned GNN. Tanimoto similarities were computed on Morgan FPs (section Molecular representations)

In order to ascertain the applicability domain of our models in the low-data regime, we examined the quality of FSL predictions in the test set *dset* across a range of distances to the training set *dtrain*. We grouped test molecules by their Tanimoto similarity to the training set and computed prediction metrics for each model within each group (Fig. 6). We compared predictions by the MG-CNP with predictions from two single task baselines (FP-GP and FP-RF), one transfer-learning baseline (fine-tuned GNN) and one meta-learning baseline (ADKF-IFT). All models used 50 molecules from *ftest, dtrain* as either training points (single-task and fine-tuning models) or context points (meta-learning models) and their predictions were evaluated on *ftest, dtest*. We observed that the MG-CNP generalized better than other models across the range of distances to the training set. Its performance was closely followed by that of the fine-tuned GNN, which stood out as a good candidate in the low-data regime which could be used in cases where uncertainty is not required, and which is perhaps simpler to implement than meta-learning alternatives. Finally, single-task models (FP-GP and FP-RF) and the ADKF-IFT displayed poor performance in test molecules distant to the training set, but achieved decent performance above 0.3 Tanimoto similarity.

It is worth noting that our applicability domain results are specific to our dataset and to the size of the context and training set used in this experiment (50). Obviously, we would not expect the MG-CNP to generalize perfectly to every region of chemical space, and neither would we expect it to outperform single-task models in a high-data setting with hundreds of thousands or millions of training datapoints. Nonetheless, our results suggest that strategies that share knowledge across tasks such as meta-learning or transfer learning may prove beneficial to the applicability domain of models for molecular property prediction in the low-data setting.

### Calibration of uncertainty estimates

**Fig. 7:**
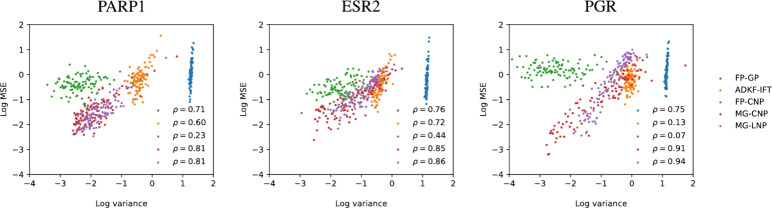
Calibration of uncertainty estimates from models in section Few-shot learning (FSL). Each point in the scatterplot indicates the log mean MSE and log mean predicted variance in each confidence percentile (percentiles of target datapoints ranked by predicted variance). The legend in each plot shows the Pearson correlation between the log MSE and the log variance for each model. The MG-CNP achieved the best correlation on the meta-test functions PARP1, ESR2 and PGR

NP predictions consist of a mean and variance value for each target point. Predictive variances can be viewed as an estimation of the confidence that the NP places on its own predictions. A model is well calibrated if, on average, lower predictive variances correlate with lower prediction errors. Figure 7 compares the calibration of MG-NPs to other models from section Few-shot learning (FSL). We selected 200 context datapoints at random (using the same data split as before, see section Dataset and data splits) and ranked the 2.5k target datapoints by predicted variance from most confident (lowest predicted variance) to most uncertain (highest predicted variance). Then, we partitioned them into 100 groups of 25 datapoints, which we call confidence percentiles; lower percentiles represent higher confidence. We computed the average mean square error (MSE) and the average predicted variance within each percentile. Figure 7 shows that the highest correlation between the predicted variance and the prediction error was achieved by the MG-CNP, closely followed by the MG-LNP.

**Fig. 8:**
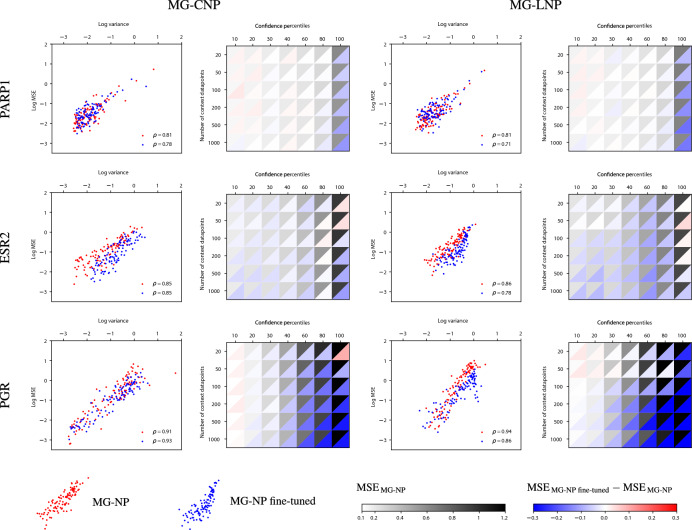
Impact of fine-tuning on uncertainty estimates. Scatterplots on the left show the log mean MSE and log mean predicted variance in each confidence percentile (percentiles of target datapoints ranked by predicted variance). The right heatmaps depict the absolute MSE of MG-NPs (black and white scale; black means higher error) and the MSE difference between the fine-tuned and the unmodified models (blue and red scale; blue indicates that fine-tuned is better)

In addition to comparing the calibration of MG-NPs and other models, we were also interested in the effect of fine-tuning on MG-NPs. Figure 8 examines the impact of fine-tuning on the uncertainty estimates, as well as calibration across different context sizes. On the left columns, the scatter plots show the log mean MSE and log mean variance of an unmodified NP (i.e. without parameter adaptation, in red) and a fine-tuned NP (in blue), using 200 random context points. The Pearson correlation between predicted variance and prediction error remains high in the fine-tuned NP ($\rho > 0.7$ in all cases), suggesting good calibration. On the right, the heatmaps show the MSE of the unmodified and fine-tuned models, using context sets ranging from 20 to 1000 datapoints. The row dimension indicates context set size and the column dimension shows test datapoints grouped by confidence percentile (the first column includes the 1^st^ to the 10^th^ percentile, the second the 11^th^ to the 20^th^, etc). In each cell, the black and white scale represents the MSE of the unmodified model. As before, we observe that MG-NPs are well calibrated, with higher errors in the higher percentiles. Note that increasing the context set size (i.e. moving from top to bottom in the heatmaps of Fig. 8) also improved performance and decreased MSE, as shown in Table 1 and in Supplementary Section D. However, the difference was small compared to the difference across confidence percentiles, so the latter dominated the black and white color scale. The red and blue scale indicates the difference between the MSE of the fine-tuned and the unmodified models. Blue cells signify that the fine-tuned model beat the unmodified one in that context size and confidence pair. In general, most fine-tuning gains came from the most erroneous unmodified predictions. This is to be expected: regions of the input space where predictions have higher loss will provide a larger signal for parameter adaptation.

Overall, our results suggest that MG-NPs are able to provide well-calibrated uncertainty estimates, and that fine-tuning for a small number of effective epochs can substantially improve mean predictions (Table 1) while maintaining good calibration (Fig. 8) without overfitting to the meta-test function.

### Randomization of context and target sets protects NPs from overfitting

The MG-CNP, a model with millions of parameters, exhibited good uncertainty calibration in meta-test functions (Figs. 7 and 8) despite having been trained with a maximum likelihood objective (Eq. 1) on as few as 2.5k molecules. Even though the CNP objective lacks a regularization term like that of the LNP, we did not find evidence that the model overfit, in the sense of underestimating posterior uncertainty. We hypothesized that this may be due to the randomization of context and target points during meta-training. The effect of this process may be two-fold: first, random sampling induces a combinatorially large number of unique views of each task or function, which may resemble an augmentation of the function set; and second, by using only a subset of all observations at each epoch, the number of effective epochs (i.e. number of times a datapoint is seen during training) is kept low, hence reducing the risk of overfitting to any single datapoint.

**Fig. 9:**
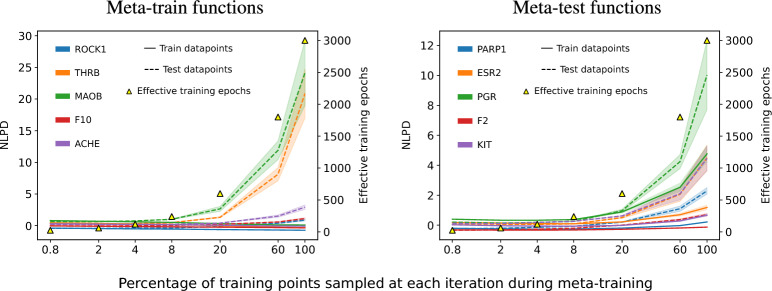
Random sampling of observations during meta-training protects from overfitting. Increasing the percentage of points sampled as contexts or targets leads to less unique views of each task and to more effective epochs. This causes overfitting, with memorization of the labels from the train points of the train functions (left, solid lines) and a degradation of performance on the test points of the train functions (left, dashed line) and all points of the test functions (right, solid and dashed lines). Lower NLPD is better

In previous experiments, we trained MG-NPs using between 0.8% and 6% of observations as contexts and targets at each iteration. To investigate if random subsampling during meta-training protects NPs from overfitting, we trained a collection of MG-CNPs with the same architecture and on the same dataset as before, but using increasing fractions of observations as contexts and targets at each iteration. (Note that in this experiment we allowed overlapping of context and target sets during meta-training in order to be able to examine context and target sizes of up to 100% of the training datapoints; otherwise, if we kept context and target sets disjoint, the maximum simultaneous context and target size would be 50%). Then, we examined the performance of these models in terms of negative log predictive density (NLPD), a measure of calibration of uncertainty estimates (lower is better). We observed that as the fraction of points sampled at each training iteration growed, MG-CNPs overfit to the training molecules of the training tasks, as evidenced by the low NLPD in these molecules (Fig. 9, left, solid lines) and high NLPD in the test molecules of the training tasks (Fig. 9, left, dashed lines) and in all molecules of the test tasks (Fig.9, right, solid and dashed lines). This loss of calibration also manifested as poor correlation between the predicted uncertainty and the prediction error (Supplementary Figure G.2). These results suggest that, in order to achieve adequate meta-generalization and calibration, it is critical to tune the size of the context and target sets during meta-training.

### Bayesian optimization (BO)

We benchmarked MG-CNP, ADKF-IFT and a GP on binary fingerprints in a sequential learning experiment using two objective functions from the DOCKSTRING optimization benchmark, druglike F2 and selective JAK2. The former encourages molecules that dock well against F2, while the latter favours molecules that dock well against JAK2 but dock poorly against LCK. Both objectives are penalized with a QED (quantitative estimate of drug-likeness) score which encourages compounds that have similar properties to orally-absorbed approved small-molecule drugs. A detailed description of the objectives is provided in Supplementary Section H. For this experiment, we created a library of 60k molecular candidates by sampling 30k from the DOCKSTRING training set and 30k from the test set. We selected molecules from the library in batches of 5 at a time, up to a total budget of 1000, using a lower confidence bound (LCB) or greedy acquisition function (which selected molecules according to the best predicted mean). For meta-training tasks, we created an augmented dataset of random combinations of transformed docking scores and QED (quantitative estimate of drug-likeness). To avoid data leakage across functions, we excluded from the augmentation procedure the proteins that participated in the objectives, i.e. F2, JAK2 and LCK. More details about the BO molecule library are provided in section Dataset and data splits.

**Fig. 10:**
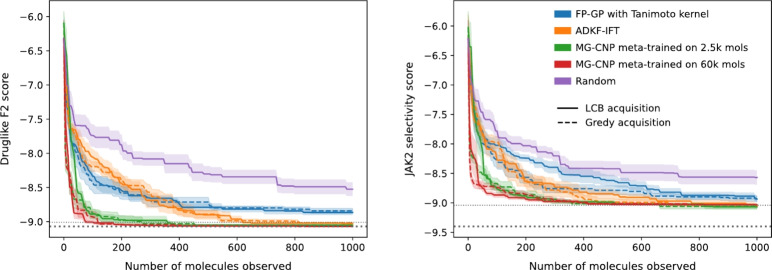
Bayesian optimization of druglike F2 (left) and selective JAK2 (right). MG-CNPs always reached either the best or a near-best molecule, outperforming ADKF-IFT and GPs with a Tanimoto kernel on binary FPs. Trajectory lines indicate the mean of 10 trajectories with different random initializations, and shaded area indicates the standard error. Horizontal dotted lines indicate the best and second-best molecules in the library of 60k compounds

We compared two MG-CNPs, one meta-trained on 2.5k molecules (Fig. 10, orange) and another meta-trained on all 60k molecules from the library (green). The two MG-CNPs displayed similar BO trajectories, suggesting that NPs could be effective for molecular optimization even in the low-data setting. In druglike F2, they often reached the best molecule in the library or another with very similar score (Fig. 10, left). In selective JAK2, they always reached the second-best molecule in the library (Fig. 10, right). As baselines, we compared to a GP with a Tanimoto kernel on binary FPs (blue), and to random selection (red). The GP performed significantly better than random but the MG-CNP always found better molecules than the GP. Interestingly, LCB acquisition (solid line) and greedy acquisition (dashed line) yielded comparable performance, both in MG-CNPs and in the GP. While LCB with a well-calibrated model should perform better on expectation (i.e. given an infinite budget and choosing from a sufficiently large library of molecules), it may not always be beneficial in every data regime.

**Fig. 11:**
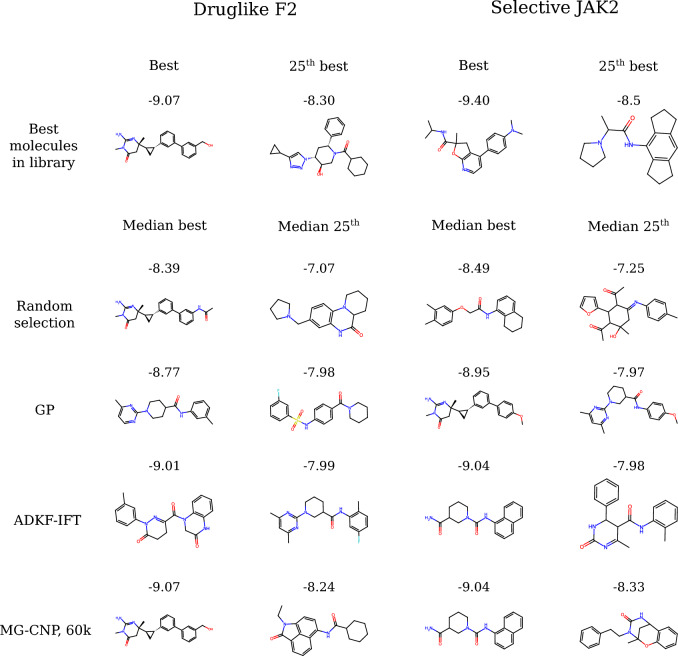
Median best and 25-th best molecules selected by each model, compared to the best and 25-th best molecules in the library. The models GP, ADKF-IFT and MG-CNP in this figure used a LCB acquisition

To ascertain whether any scaffolds or groups predominated or were favoured by the models, we visualized the median best molecule (i.e. the 5-th best within the 10 best molecules from the 10 experiment replicates) and the median $25^{\text {th}}$ molecule (i.e. the $5^{\text {th}}$ best molecule within the 10 25-th best molecules from the 10 experiment replicates) selected by each model for each objective function. We did not observe any obvious preference or bias, suggesting that MG-CNPs were able to adapt their predictions to the molecules in the library to find optimal candidates (Fig. 11).

## Limitations and future work

In this paper, we have presented an evaluation of meta-learning with NPs for molecular property prediction. We have performed our benchmarking experiments on a subset of the DOCKSTRING dataset of docking scores, which includes scores against 58 protein targets computed with AutoDock Vina. While the field of meta-learning for chemistry is gaining attention [4, 13–17, 39], many questions remain open about the application of meta-learning to real-world datasets, particularly with regards to data splitting (across tasks and datapoints) and to the minimum number of labels required for meta-learning to become effective. In addition, one of the advantages of NPs is that they provide uncertainty estimates; the calibration of these must be analyzed. A simulated property like docking scores was suitable to investigate these aspects because it allowed labelling arbitrary molecules and creating an optimal benchmarking dataset. In particular, the DOCKSTRING dataset enabled studying or controlling the following methodological features:

- Meta-generalization: full datapoint overlap across protein targets in DOCKSTRING enabled quantifying the distance between proteins as the Pearson correlation between their docking scores. This way, we could identify which test tasks were close or far away from the training tasks, which helped us develop parameter adaptation strategies for highly-divergent tasks.
- Molecule split: datapoint overlap allowed re-using the datapoint split in all tasks so that the same datapoints served as either contexts or targets in every task. This minimized the risk of data leakage from one task to another. In addition, we split molecules by cluster, which minimized the risk of data leakage within tasks.
- Context size: since we could choose the desired size of the DOCKSTRING subset in our experiments, we were able to compare the performance of single-task, transfer-learning and meta-learning methods over a range of context sizes (from 20 to 1000, section Few-shot learning (FSL)). We were also able to observe the increasing impact of fine-tuning as the number of contexts grew.
- Uncertainty calibration: predicted variance may be indicative of the true error on average but it cannot indicate the true error of each individual datapoint. Therefore, having many labelled target molecules available (2.5k in our DOCKSTRING subset) facilitated studying uncertainty calibration in section Calibration of uncertainty estimates. In particular, we grouped target points by percentiles of predicted variance and computed the average predicted variance and average error within each percentile.

These aspects, which are essential for methodological development and benchmarking, cannot be accurately controlled in experimental bioactivity datasets because they usually have few and non-overlapping labels. However, imputation of sparse datasets and bioactivity prediction are two areas where meta-learning could make a great impact. Therefore, although our work represents a significant step towards the practical deployment of NPs for molecules, restricting our experiments to simulated data is an important limitation of our study. We leave the application to real bioactivity measurements as future work. Real bioactivity datasets will require careful examination of the intra-task heterogeneity [17, 23], since in order for meta-learning to be effective in a certain task, the labels in that task should be self-consistent. For example, we do not expect meta-learning to be helpful in predicting a task which combines measurements from two different assays against the same protein, if we employ the labels from one assay as contexts and the labels from the other assay as targets. This situation of combining different assays as a single task is a common source of noise in public bioactivity databases [23].

Another future area of research with high potential for molecular-property prediction in the low-data setting is the utilization of sequence models and large language models (LLMs) as foundation models that could be fine-tuned to novel tasks. Sequence models trained on SMILES in an unsupervised manner can produce highly informative molecular embeddings that outcompete molecular fingerprints [40]. Surprisingly, similar results have been reported for embeddings derived from general-purpose LLMs trained on natural language, even though were they were not exposed to chemical tasks [41]. These large models could be treated as molecular featurizers, and their embeddings could be further improved by parameter adaptation with specialized techniques [42].

## Discussion

Molecular property prediction poses a unique challenge to meta-learning algorithms such as NPs due to the inherent heterogeneity of molecular tasks. Using docking scores against a variety of protein targets from different families, we created a benchmarking dataset where we could control the amount of datapoints available for learning, and where we could objectively quantify the similarity or divergence between tasks in terms of the correlation of their docking scores. This controlled environment allowed us to evaluate predictions across a range of context sizes, and to investigate parameter adaptation strategies to improve meta-generalization to heterogeneous test functions. We used our benchmark to evaluate a novel NP architecture on molecular graphs, which we called the MG-NP.

Our large-scale benchmark demonstrates that MG-NPs outperform a variety of baselines in FSL, including single-task and traditional transfer-learning approaches for neural networks, probably due to the fact that they can efficiently transfer knowledge across many similar tasks simultaneously in a principled manner. In addition, we propose fine-tuning strategies which address one of the main shortcomings of NPs—the inflexible way in which training points are used in meta-testing—showing that they significantly improve meta-generalization. Fine-tuning proved highly beneficial when predicting docking scores of divergent test proteins such as PGR.

NPs are particularly attractive because they perform uncertainty quantification. We find that, despite the difference in their training loss function, both MG-CNPs and MG-LNPs provide well-calibrated uncertainty estimates, and these estimates do not deteriorate with fine-tuning. Furthermore, we show that randomization of context and target sets, and an adequate tuning of their size, are critical to calibration. Finally, we demonstrate the use of meta-learned MG-CNPs in a Bayesian optimization experiment, outperforming both more traditional models such as GPs on binary fingerprints, and other meta-learning models such as ADKF-IFT.

Overall, our work presents a novel GNN architecture for NPs, the MG-NP, that is competitive against a variety of baselines, and we suggest strategies for meta-training and meta-testing that increase the applicability of NPs for molecular property prediction.

## Supplementary Information

Supplementary Material 1.

## Data Availability

The source code is available from https://github.com/mgarort/graph-nps-for-mols.
