# Supplementary material for: Graph neural processes for molecules: an evaluation on docking scores and strategies to improve generalization
Source: J Cheminform. 2024 Oct 23;16:115. doi: 10.1186/s13321-024-00904-2 (PMC11515514; doi:10.1186/s13321-024-00904-2)
Supplement: Supplementary file 1 — Supplementary Material 1. [file 13321_2024_904_MOESM1_ESM.pdf]

Supplementary information for

"Graph neural processes for molecules: An evaluation on docking scores and strategies to improve meta-generalization"

A Neural processes (NPs)

A.1 Parameterization of CNP and LNP

NPs assume conditional independence between the targets and a Gaussian predictive distribution with mean $\mu_\theta(x)$ and variance $\sigma_\theta^2(x)$:

$$q\left(y_t \mid x_c, y_c\, ; x_t\right) = \prod_{j=1}^{T} \mathcal{N}\left(y_{t,j}\, ;\, \mu_\theta\left(x_{t,j}, x_c, y_c\right), \sigma_\theta^2\left(x_{t,j}, x_c, y_c\right)\right).$$

The conditional NP (CNP) [1] and the latent NP (LNP) [2] parameterize the predictive mean and variance of an input x in three steps:

- *Datapoint encoder*: a neural network h_θ maps each context point $(x_{c,j}, y_{c,j})$ to a local datapoint representation r_j.

$$r_j = h_\theta\left(x_{c,j}, y_{c,j}\right)$$

- *Function encoder*: all context encodings r_j are combined into a global context encoding r through a commutative operation $\oplus$, usually the sum or the mean. Commutativity of the function encoder guarantees invariance to permutations of the context set.

$$r = r_1 \oplus \cdots \oplus r_C$$

- *Decoder*: a neural network g_θ maps the function encoding r and the input location x to the predictive mean and variance. In the CNP, the decoding process is deterministic:

$$\left(\mu_\theta(x, x_c, y_c), \sigma_\theta^2(x, x_c, y_c)\right) = g_\theta\left(r, x\right).$$

  In the LNP, the decoding process involves sampling a latent variable. Its approximate posterior q_ϕ is parameterized as a Gaussian with mean and variance given by a neural network g_ϕ.

$$z \sim q_\phi(z \mid x_c, y_c) := \mathcal{N}\left(\mu_\phi, \sigma_\phi^2\right), \quad \left(\mu_\phi, \sigma_\phi^2\right) = g_\phi(r)$$

The final decoding stage continues like the CNP, but using z instead of r:

$$\left(\mu_\theta(x, x_c, y_c), \sigma_\theta^2(x, x_c, y_c)\right) = g_\theta(z, x).$$

A.2 Objective functions of neural processes (NPs)

The CNP objective $\mathcal{L}_\theta$ is the conditional log likelihood of the targets given the contexts [1].

$$\mathcal{L}_\theta(y_t \mid x_c, y_c; x_t) = \log q_\theta(y_t \mid x_c, y_c; x_t)$$

The LNP objective $\mathcal{L}_{\theta,\phi}$ consists of a reconstruction and a regularization term [2]:

$$\mathcal{L}_{\theta,\phi}(y_t \mid x_c, y_c; x_t) = \mathbb{E}_{q_\phi(z \mid x_d, y_d)}\left[\log p_\theta(y_t \mid z, x_t)\right] - D_{\mathrm{KL}}\left(q_\phi(z \mid x_d, y_d) \,\|\, q_\phi(z \mid x_c, y_c)\right),$$

where $x_d = (x_c, x_t)$, $y_d = (x_c, y_t)$ and D_{KL} is the KL divergence. It is an approximation of an evidence lower bound (ELBO) to the conditional log marginal likelihood:

$$\log p_\theta(y_t \mid x_d, y_c) \geq \mathbb{E}_{q_\phi(z \mid x_d, y_d)}\left[\log p_\theta(y_t \mid z, x_t)\right] - D_{\mathrm{KL}}\left(q_\phi(z \mid x_d, y_d) \,\|\, p_\theta(z \mid x_c, y_c)\right).$$

The ELBO is intractable due to the posterior $p_\theta(z \mid x_c, y_c)$ in the KL term. The LNP objective is derived by approximating the posterior with $q_\phi(z \mid x_c, y_c)$. This way the objective becomes tractable but $\mathcal{L}_{\theta,\phi}$ is no longer an analytical lower bound.

B Number of observations per task in bioactivity datasets

FSL experiments in other domains (e.g. image classification) often focus on an extremely low number of context points, rarely benchmarking more than 100 contexts. In the context of drug discovery, however, the amount of observations per function in bioactivity datasets can vary widely, with many functions of interest having more than 100 labels. For example, in Figure B.1 we show the distribution of observations registered in protein binding affinity assays in ChEMBL33 [3]. Whether meta-learning can provide benefits in this data regime is an open research question. For this reason, we decided to investigate a wide range of context points, from 20 to 1000 (Table 1 and Supplementary Section D).

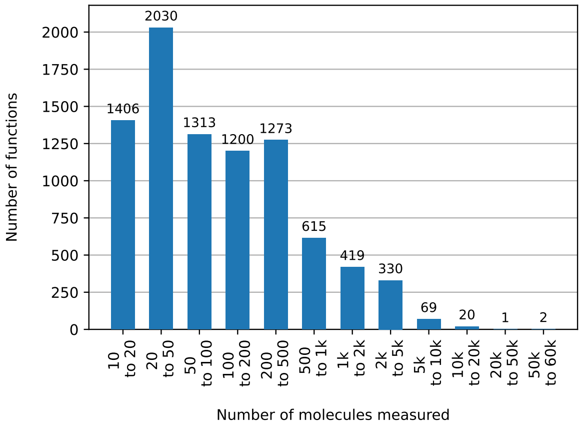

Fig. B.1: Distribution of observations per function in the protein binding subset of ChEMBL33

C Few-shot learning with MAML

C.1 Parameter adaptation with MAML

Model-agnostic meta-learning (MAML) [4] is a meta-training approach to find model parameters that can be rapidly adapted to test functions in one or more steps of gradient-descent. At each meta-training iteration t, MAML simulates an adaptation experiment in two stages: the inner update (the simulated adaptation) and the outer update (the actual update of the model's parameters). First, during the inner update, it samples a set of support points from a test function, computes the loss of their predictions (inner loss), and takes a single step of gradient descent to adapt the model's parameters from θ_t to θ'_t. Second, during the outer update, MAML samples another set of query points from the same function, computes the prediction loss on those points using θ'_t (outer loss), and backpropagates through the inner update to find new parameters θ_{t+1}. Since the inner update involves a step of gradient descent, the outer update involves computing a gradient through a gradient, i.e. computing the Hessian of the parameters.

Similar to the support and query sets in MAML, meta-training NPs entails sampling two sets of observations: the contexts and the targets. The support set in MAML informs the prediction of the query set, analogous to how the context set in NPs informs the prediction of targets. We can meta-train NPs with MAML by computing the inner loss on the context points and the outer loss on the target points. At each iteration t, during the inner update a prediction on the contexts of a train function f_i is made, with loss $\mathcal{L}_{\psi_t}(y^i_c \mid x^i_c, y^i_c\,; x^i_c)$. A single step of gradient descent is taken to adapt the parameters from ψ_t to ψ'_t. During the outer update, a prediction on the targets is made using the adapted parameters ψ'_t, with loss $\mathcal{L}_{\psi'_t}(y^i_t \mid x^i_c, y^i_c\,; x^i_t)$. Finally, we backpropagate through the inner update to find new parameters ψ_{t+1}. Later, at meta-testing, we adapt NPs by taking a single iteration of gradient descent on the loss of the contexts of f, $\mathcal{L}_{\psi}(y_c \mid x_c, y_c\,; x_c)$.

Computing the Hessian of all MG-NP parameters was memory-prohibitive, so we only applied MAML to some layers, similar to Raghu et al. [5]. In particular, we applied it to the same layers adapted in the fine-tuning experiments. During meta-training, these layers underwent the inner and outer update cycle, while other layers experienced a single update per iteration, as usual. Later, during meta-testing, the MAML layers were adapted and the rest were frozen. In addition, backpropagating through gradients can lead to training instability, so we implemented modifications from Antoniou et al. [6] to increase robustness.

C.2 MAML implementation

In all experiments involving MAML (whether single-task GNN or NPs), we used a single step of gradient descent in the inner update with a learning rate of 10^{-3}. We attempted other rates to try to increase training stability and decrease MAML's error bars but found this to be the most stable. In addition, our MAML training regime borrowed tricks from Antoniou et al. [6] to improve robustness. In particular, we implement derivative-order annealing such that the first 50 epochs of training do

first-order MAML and the rest do second-order MAML. We also implement cosine annealing of the outer learning rate. The tricks of multi-step loss optimization and per-step batch normalization are not applicable to our model since we performed a single gradient descent iteration in the inner loop. Due to memory constraints, we follow Raghu et al. [5] and only apply MAML the last two layers of the encoder or the decoder of NPs, as explained in Section 3.4. Similarly, we only train the last two layers of single-task GNNs meta-trained with MAML.

C.3 MAML few-shot learning results

Few-shot learning (FSL) results for models trained with a MAML regime are shown in the extended FSL results (Supplementary Section D).

D Extended few-shot learning (FSL) results

		PARP1 (high correlation)						ESR2 (medium correlation)						PGR (low correlation)					
		Number of context points						Number of context points						Number of context points					
		20	50	100	200	500	1000	20	50	100	200	500	1000	20	50	100	200	500	1000
Single task	Dummy regressor	-13.6 (3.9)	-18.9 (3.0)	-19.6 (2.9)	-21.4 (1.7)	-21.9 (1.1)	-21.4 (0.6)	-5.5 (1.9)	-3.5 (1.1)	-3.0 (1.1)	-3.5 (0.8)	-3.3 (0.4)	-3.1 (0.3)	-13.3 (4.6)	-5.3 (1.9)	-2.7 (0.8)	-1.6 (0.4)	-1.6 (0.3)	-1.5 (0.2)
	FP-RF	0.4 (2.1)	-1.2 (2.3)	3.0 (2.5)	15.1 (1.9)	23.7 (0.8)	30.9 (0.5)	-5.3 (3.0)	6.6 (2.0)	11.2 (1.4)	15.4 (1.4)	21.0 (0.5)	25.1 (0.2)	-13.2 (8.7)	-4.7 (2.2)	-0.3 (1.4)	2.0 (1.3)	**8.9 (0.8)**	11.9 (0.5)
	FP-GP	6.4 (0.7)	9.5 (1.7)	15.6 (2.1)	29.6 (1.3)	47.5 (0.7)	58.1 (0.3)	**2.0 (1.0)**	**8.3 (1.3)**	**14.0 (1.3)**	**19.9 (0.9)**	**26.7 (0.4)**	**31.8 (0.4)**	**-11.3 (4.9)**	**1.0 (1.8)**	**6.2 (0.9)**	**8.4 (0.7)**	**8.9 (0.6)**	11.2 (0.8)
	GNN	**22.9 (11.9)**	**21.0 (13.9)**	**63.9 (4.2)**	**61.7 (9.0)**	**73.2 (0.6)**	**75.4 (0.5)**	-10.7 (4.9)	-7.4 (3.6)	1.8 (3.9)	6.4 (3.9)	14.8 (3.5)	19.8 (4.2)	-56.6 (33.0)	-13.0 (6.6)	-4.4 (2.6)	-1.9 (4.8)	6.7 (1.6)	**17.4 (3.0)**
Transfer learning	GNN (random)	63.8 (1.4)	69.9 (1.3)	70.7 (2.5)	73.7 (1.3)	76.2 (0.7)	77.6 (1.3)	5.7 (3.2)	19.1 (2.1)	10.8 (4.9)	17.8 (2.5)	24.3 (2.0)	29.2 (1.6)	-21.2 (16.9)	14.5 (2.6)	9.9 (3.8)	13.5 (3.9)	**23.3 (1.6)**	**29.9 (0.7)**
	GNN (fine-tuned)	**75.0 (0.9)**	**77.3 (0.6)**	**77.9 (0.3)**	**76.2 (0.4)**	**75.4 (0.3)**	**77.1 (0.2)**	**34.9 (1.5)**	**37.8 (0.8)**	**35.2 (1.1)**	**30.3 (1.2)**	**30.3 (1.0)**	**34.2 (0.6)**	**21.1 (1.8)**	**26.4 (1.2)**	**23.7 (1.4)**	**16.3 (1.1)**	19.4 (1.0)	24.7 (1.0)
Meta-learning	GNN (MAML)	51.6 (8.9)	52.2 (9.0)	52.0 (9.0)	51.0 (9.8)	50.9 (9.8)	51.3 (9.8)	11.5 (11.1)	12.7 (11.0)	11.3 (10.5)	10.8 (10.3)	9.8 (10.4)	11.1 (10.6)	-31.2 (7.7)	-26.6 (6.2)	-28.4 (6.3)	-30.5 (6.4)	-32.4 (6.8)	-33.0 (6.4)
	ADKF-IFT	10.8 (3.4)	28.2 (1.8)	38.8 (1.5)	47.5 (1.1)	55.2 (0.6)	61.9 (0.3)	-0.3 (1.4)	8.1 (1.3)	13.9 (0.9)	18.0 (1.1)	24.6 (0.7)	29.4 (0.4)	-6.6 (4.1)	1.7 (1.4)	4.5 (0.7)	5.2 (0.8)	8.7 (0.9)	14.6 (0.4)
	FP-CNP	55.5 (0.7)	55.5 (0.5)	55.4 (0.5)	55.2 (0.4)	55.4 (0.3)	55.3 (0.4)	29.0 (0.8)	26.0 (2.0)	27.2 (1.3)	28.8 (0.7)	29.1 (0.6)	29.7 (0.5)	-36.7 (4.7)	-37.5 (4.5)	-39.1 (3.8)	-39.7 (2.3)	-42.4 (2.9)	-41.6 (2.5)
	MG-CNP	**81.6 (0.6)**	**82.7 (0.3)**	**83.0 (0.1)**	**83.1 (0.1)**	**83.1 (0.1)**	**83.0 (0.2)**	**43.2 (1.0)**	**44.9 (0.7)**	**45.3 (0.6)**	**45.6 (0.5)**	**46.3 (0.5)**	**46.6 (0.6)**	**19.1 (2.5)**	**23.2 (1.7)**	**25.6 (1.4)**	**27.5 (1.5)**	**27.5 (1.2)**	**27.2 (1.3)**
	MG-LNP	81.4 (0.3)	81.5 (0.3)	81.6 (0.3)	81.7 (0.3)	82.0 (0.3)	82.3 (0.3)	39.9 (1.3)	40.0 (1.3)	40.1 (1.2)	40.3 (1.2)	41.0 (1.0)	41.5 (0.9)	2.4 (3.7)	3.0 (3.7)	3.8 (3.7)	5.5 (3.8)	10.6 (3.7)	15.7 (3.4)
NPs with parameter adaptation	FP-CNP (MAML)	57.8 (0.5)	58.0 (0.6)	58.3 (0.4)	58.2 (0.3)	58.2 (0.3)	58.3 (0.3)	25.8 (2.6)	24.5 (2.0)	24.7 (1.4)	26.7 (1.3)	26.8 (1.0)	27.9 (0.7)	-26.4 (6.0)	-19.0 (2.3)	-19.0 (1.8)	-20.7 (1.6)	-20.2 (1.6)	-19.9 (1.6)
	FP-CNP (fine-tuned)	55.3 (0.8)	55.2 (0.7)	55.7 (0.5)	55.7 (0.4)	56.1 (0.3)	56.2 (0.2)	30.4 (0.8)	29.5 (1.2)	29.9 (0.9)	31.4 (0.7)	33.3 (0.3)	34.0 (0.4)	-24.5 (5.2)	-13.7 (1.5)	-9.2 (1.3)	-5.7 (1.5)	1.6 (1.1)	7.7 (0.8)
	FP-LNP (MAML)	42.1 (0.6)	42.3 (0.8)	42.5 (0.7)	42.4 (0.7)	42.5 (0.8)	42.4 (0.8)	6.8 (5.4)	8.0 (4.6)	8.2 (4.2)	6.9 (4.3)	7.4 (4.2)	7.3 (4.2)	-24.1 (7.9)	-21.2 (6.7)	-21.0 (6.4)	-21.6 (6.5)	-21.3 (6.3)	-20.9 (6.3)
	FP-LNP (fine-tuned)	38.8 (1.3)	36.0 (1.4)	37.6 (1.4)	40.3 (1.2)	41.6 (1.2)	41.2 (0.7)	29.6 (0.6)	30.6 (0.6)	29.8 (0.5)	29.0 (0.8)	30.4 (0.6)	29.8 (0.7)	-1.2 (3.4)	3.6 (0.6)	7.7 (0.8)	8.3 (0.6)	7.3 (0.9)	6.2 (0.6)
	MG-CNP (MAML)	82.1 (0.5)	83.3 (0.3)	83.5 (0.2)	83.7 (0.2)	83.7 (0.2)	83.7 (0.2)	44.0 (0.9)	44.9 (0.9)	44.8 (1.2)	45.9 (0.5)	46.7 (0.5)	47.0 (0.4)	**22.2 (2.2)**	29.7 (1.5)	31.3 (1.6)	32.6 (1.4)	33.3 (1.1)	33.4 (1.2)
	MG-CNP (fine-tuned)	82.1 (0.5)	**83.4 (0.2)**	**83.7 (0.2)**	**83.9 (0.2)**	**84.4 (0.2)**	**84.9 (0.1)**	**45.0 (0.9)**	**45.3 (0.8)**	**46.9 (0.8)**	**49.7 (0.4)**	**51.3 (0.4)**	**52.5 (0.4)**	20.3 (3.9)	**31.9 (1.4)**	**36.6 (1.2)**	**40.7 (0.8)**	**43.7 (0.2)**	**45.1 (0.3)**
	MG-LNP (MAML)	80.8 (0.6)	80.7 (0.6)	80.8 (0.6)	80.9 (0.6)	81.2 (0.5)	81.5 (0.5)	38.4 (1.3)	39.5 (0.9)	39.9 (0.9)	39.9 (0.9)	40.5 (0.8)	41.1 (0.7)	10.2 (3.1)	11.5 (2.8)	12.2 (2.7)	13.2 (2.7)	16.0 (2.4)	19.7 (1.9)
	MG-LNP (fine-tuned)	**82.5 (0.4)**	82.3 (0.5)	83.0 (0.3)	83.5 (0.3)	84.2 (0.2)	84.7 (0.2)	43.3 (0.9)	42.8 (0.9)	46.2 (0.6)	47.3 (0.7)	49.5 (0.4)	50.4 (0.6)	10.8 (9.3)	7.7 (6.5)	18.3 (3.7)	31.6 (2.0)	40.1 (1.5)	42.2 (1.6)

Table D.1: Coefficient of determination (R^2) few-shot learning results for all models, including those trained with MAML (Section C). Each cell shows the mean and standard error (in parentheses) of 10 random repetitions.

D Extended few-shot learning (FSL) results

		PARP1 (high correlation)						ESR2 (medium correlation)						PGR (low correlation)					
		Number of context points						Number of context points						Number of context points					
		20	50	100	200	500	1000	20	50	100	200	500	1000	20	50	100	200	500	1000
Single task	FP-GP	**0.85 (0.01)**	**0.71 (0.01)**	**0.64 (0.01)**	**0.55 (0.01)**	**0.40 (0.01)**	**0.28 (0.00)**	**0.52 (0.01)**	**0.40 (0.01)**	**0.35 (0.01)**	**0.31 (0.00)**	**0.26 (0.00)**	**0.22 (0.00)**	**0.64 (0.04)**	**0.49 (0.02)**	**0.43 (0.01)**	**0.41 (0.01)**	**0.40 (0.00)**	**0.38 (0.00)**
Meta-learning	ADKF-IFT	**0.73 (0.04)**	**0.57 (0.02)**	**0.48 (0.02)**	**0.39 (0.01)**	**0.31 (0.01)**	**0.22 (0.00)**	**0.41 (0.01)**	**0.36 (0.01)**	**0.32 (0.00)**	**0.29 (0.01)**	**0.25 (0.00)**	**0.21 (0.00)**	**0.52 (0.03)**	**0.44 (0.01)**	**0.42 (0.01)**	**0.41 (0.01)**	**0.40 (0.01)**	**0.36 (0.00)**
	FP-CNP	3.61 (0.11)	3.50 (0.11)	3.52 (0.07)	3.49 (0.06)	3.49 (0.05)	3.50 (0.06)	1.69 (0.06)	1.86 (0.10)	1.76 (0.07)	1.65 (0.04)	1.63 (0.04)	1.60 (0.04)	7.29 (1.16)	8.03 (1.17)	7.83 (0.79)	7.44 (0.45)	8.16 (0.58)	8.03 (0.44)
	MG-CNP	0.83 (0.06)	0.78 (0.02)	0.75 (0.01)	0.74 (0.01)	0.74 (0.01)	0.74 (0.01)	1.04 (0.01)	1.00 (0.01)	0.98 (0.01)	0.98 (0.01)	0.97 (0.01)	0.97 (0.01)	1.18 (0.02)	1.13 (0.02)	1.09 (0.01)	1.07 (0.01)	1.07 (0.01)	1.08 (0.01)
	MG-LNP	0.75 (0.01)	0.75 (0.01)	0.75 (0.01)	0.75 (0.01)	0.74 (0.01)	0.74 (0.01)	1.08 (0.01)	1.07 (0.01)	1.07 (0.01)	1.07 (0.01)	1.06 (0.01)	1.05 (0.01)	1.28 (0.03)	1.27 (0.03)	1.27 (0.03)	1.25 (0.03)	1.21 (0.03)	1.16 (0.03)
NPs with parameter adaptation	FP-CNP (MAML)	3.42 (0.17)	3.35 (0.12)	3.31 (0.08)	3.27 (0.06)	3.18 (0.05)	3.19 (0.03)	1.69 (0.10)	1.77 (0.13)	1.70 (0.10)	1.60 (0.05)	1.57 (0.03)	1.53 (0.02)	4.60 (0.52)	5.18 (0.49)	5.13 (0.40)	5.09 (0.29)	5.23 (0.26)	5.31 (0.23)
	FP-CNP (fine-tuned)	3.76 (0.84)	3.20 (0.25)	3.46 (0.27)	3.49 (0.22)	3.46 (0.10)	3.48 (0.11)	1.18 (0.01)	1.20 (0.02)	1.16 (0.01)	1.16 (0.01)	1.17 (0.01)	1.21 (0.01)	2.36 (0.28)	2.23 (0.15)	1.69 (0.04)	1.49 (0.01)	1.45 (0.01)	1.44 (0.01)
	FP-LNP (MAML)	1.44 (0.02)	1.43 (0.02)	1.43 (0.02)	1.43 (0.02)	1.43 (0.02)	1.43 (0.02)	1.32 (0.04)	1.30 (0.03)	1.30 (0.03)	1.31 (0.03)	1.31 (0.03)	1.31 (0.03)	1.49 (0.04)	1.46 (0.03)	1.46 (0.03)	1.46 (0.03)	1.46 (0.03)	1.46 (0.03)
	FP-LNP (fine-tuned)	2.14 (0.08)	1.87 (0.02)	2.68 (0.05)	3.24 (0.09)	3.52 (0.09)	3.66 (0.11)	1.21 (0.02)	1.15 (0.00)	1.20 (0.01)	1.22 (0.01)	1.27 (0.01)	1.34 (0.01)	1.37 (0.02)	1.34 (0.01)	1.32 (0.01)	1.32 (0.00)	1.46 (0.02)	1.62 (0.03)
	MG-CNP (MAML)	0.80 (0.03)	**0.75 (0.02)**	**0.73 (0.01)**	**0.72 (0.00)**	**0.71 (0.00)**	0.71 (0.00)	1.03 (0.01)	1.00 (0.01)	0.99 (0.01)	0.98 (0.01)	0.97 (0.01)	0.96 (0.01)	**1.14 (0.02)**	**1.06 (0.01)**	1.04 (0.01)	1.03 (0.01)	1.02 (0.01)	1.02 (0.01)
	MG-CNP (fine-tuned)	0.95 (0.08)	0.84 (0.05)	0.79 (0.03)	0.76 (0.01)	0.73 (0.01)	0.70 (0.01)	**0.98 (0.01)**	**0.95 (0.01)**	**0.94 (0.01)**	**0.91 (0.01)**	**0.89 (0.01)**	**0.87 (0.01)**	1.15 (0.02)	**1.06 (0.01)**	**1.03 (0.01)**	**1.00 (0.01)**	**0.98 (0.01)**	**0.96 (0.01)**
	MG-LNP (MAML)	**0.78 (0.02)**	0.78 (0.02)	0.78 (0.02)	0.78 (0.02)	0.77 (0.02)	0.76 (0.02)	1.05 (0.01)	1.04 (0.01)	1.04 (0.01)	1.04 (0.01)	1.03 (0.00)	1.02 (0.01)	1.20 (0.02)	1.19 (0.02)	1.19 (0.02)	1.18 (0.02)	1.16 (0.02)	1.14 (0.02)
	MG-LNP (fine-tuned)	0.91 (0.07)	0.81 (0.03)	0.77 (0.02)	0.74 (0.01)	0.72 (0.01)	**0.69 (0.01)**	1.04 (0.02)	1.01 (0.01)	0.96 (0.01)	0.95 (0.01)	0.92 (0.01)	0.91 (0.01)	1.30 (0.11)	1.29 (0.07)	1.22 (0.05)	1.06 (0.01)	1.01 (0.01)	0.98 (0.01)

1 2 3 | 0.25 0.50 0.75 1.00 1.25 1.50 1.75 | 2 4 6 8

Table D.2: Negative log predictive likelihood (NLPD) few-shot learning results for all probabilistic models, including those trained with MAML (Section C). Each cell shows the mean and standard error (in parentheses) of 10 random repetitions.

E Model implementation

In every experiment we trained every model 10 times with different random initializations. We report the mean and standard error of these repetitions. The number of contexts in FSL experiments, as well as the initial points in the BO experiment, were also sampled randomly in each repetition.

The subset of points used for training each type of model (single task, transfer learning and meta-learning) is detailed in Section 3.6.

All neural models were implemented in Pytorch [7] and trained with the Adam optimizer using a learning rate of 10^{-3}, weight decay of 10^{-5} and a cosine annealing scheduler.

E.1 Single-task models

- *Random forest (RF).* Random forest regressors were implemented in scikit-learn [8] using the default parameters.
- *Gaussian process (GP).* We implemented exact GPs in GPytorch [9], using binary fingerprints and a Tanimoto kernel. The noise parameter was adjusted automatically in GPytorch by evidence maximization for 2000 epochs. Automatic relevance determination (ARD) to find an optimal lengthscale for each feature was attempted but eventually rejected, since the large number of fingerprint features led to gross overfitting.
- *Graph neural networks (GNNs).* Our GNN is a 6-layer fully-connected neural network (FNN) on top of the the MGAE, with maximum layer width of 1000 hidden neurons and layer normalization between each layer. We chose 6 layers to promote a fair comparison to our MG-NP models, which had an encoder and decoder of 3 layers each. GNNs were trained for 1000 epochs with a batch size of 50.

E.2 Transfer-learning models

- *GNN with random initialization (GNN random).* This model had the same architecture as the GNN in the previous section, but instead of training all layers, the GNN was initialized with random weights and only the last two layers were trained for 1000 epochs, while the rest were kept frozen, in a way reminiscent of a transfer learning experiment. In this way, the randomly-initalized layers worked as feature extractors.
- *GNN pre-trained and fine-tuned (GNN fine-tuned).* This model had the same architecture as the GNN but was pre-trained as a multi-task model on the 53 meta-train proteins. Then, the last layer was changed (to make it single-task) and the last two layers were trained for 1000 epochs while the rest were kept frozen.

E.3 Meta-learning models

E.3.1 Neural processes

- *Conditional neural processes (CNPs).* The FP-CNP had an encoder and decoder FNN of 3 layers each, with a maximum layer width of 1000, layer normalization between every layer, and an encoding size of 250. Layer normalization was chosen instead of batch normalization because, due to memory constraints, our batch size was just 2 functions. We choose the mean as commutative operation. The MG-CNP had the same architecture but built its encoder on top of the MGAE.
- *Latent neural processes (LNPs).* The FP-LNP had the same encoder and decoder as the CNP, and an additional network to parameterize the mean and variance of the latent variable. This network was also a FNN of 3 layers with layer normalization. The size of the latent variable was 250. Again, we chose the mean as the commutative operation for aggregation. The MG-LNP had the same architecture but it built its encoder on top of the MGAE.
- *Meta-training.* We trained using between 20 and 150 contexts and targets at each iteration. The amount of contexts and targets was itself sampled uniformly between 20 and 150. The contexts and targets were always disjoint, with the only exception of the experiment in Section 4.4 where they were allowed to overlap in order to be able to increase the size of both contexts and targets to 100% of the training set, thus increasing the number of effective epochs. In the FSL experiments, we trained for 3000 epochs on the 53 train proteins with a batch size of 2 functions per batch. In the BO experiments we trained for 1000 epochs on the augmented train set of combinations of docking scores with a batch size of 8.
- *Adaptation during meta-testing.* The adaptation procedures with fine-tuning and MAML and fine-tuning are described in Sections 3.4 and C respectively.
- *Loss functions.* As explained in Sections 3.1 and A.2, the parameters of the CNP $\psi = \{\theta\}$ were trained by backpropagation from the predictive log-likelihood

  $$\mathcal{L}_\psi(y_t \mid x_c, y_c\,; x_t) = \log q_\theta(y_t \mid x_c, y_c\,; x_t).$$

  The parameters of the LNP $\psi = \{\theta, \phi\}$ were trained by backpropagation from a loss function consisting of the predictive log-likelihood and a KL regularization term,

  $$\mathcal{L}_\psi(y_t \mid x_c, y_c\,; x_t) = \log q_\theta(y_t \mid x_c, y_c\,; x_t) + D_{\mathrm{KL}}\big(q_\phi(z \mid x_d, y_d) \,\|\, q_\phi(z \mid x_c, y_c)\big).$$

  Due to the extra regularization term, achieving convergence with LNPs is more challenging than with CNPs. In order to facilitate convergence, we tried training LNPs either with an exact KL divergence term or with an approximate one, and retained the best-performing model out of the two. We approximated the KL term

  $$D_{\mathrm{KL}}\big(q_\phi(z \mid x_d, y_d) \,\|\, q_\phi(z \mid x_c, y_c)\big) = \mathbb{E}_{q_\phi(z|x_d, y_d)}\left[\log \frac{q_\phi(z \mid x_d, y_d)}{q_\phi(z \mid x_c, y_c)}\right]$$

with Monte Carlo, taking a single sample from $q_\phi(z \mid x_d, y_d)$. We found that the FP-LNP achieved better performance when trained with an approximate KL regularization term, whereas the MG-LNP achieved better performance when trained with an exact one.

E.3.2 ADKF-IFT

We used the ADKF-IFT model with regression weights, trained and shared by the ADKF-IFT authors at https://figshare.com/articles/online_resource/adkf-ift-weights_zip/22070105 .

F The challenge of meta-generalization in sinusoids

In this experiment, we investigated the ability of meta-learning techniques to meta-generalize to 1D sinusoids that were slightly different from the ones seen during meta-training. We trained two models, a 6-layer fully-connected NN with MAML, and a fully-connected CNP with 3 encoder layers and 3 decoder layers (for implementation details, see Supplementary Section E). Sinusoids had functional form

$$y = A \sin\big(\rho\,(x - B)\,\big),$$

where A is the amplitude, ρ the frequency and B the shift. As meta-training set, we used 10^4 functions with $A \in [0.1, 5)$, $\rho = 1.0$ and $B \in [0, \pi)$. All parameters were sampled uniformly from these ranges. For each function, we sampled points uniformly in $x \in [-5, 5]$ as contexts and targets. The context and target sets' sizes ranged between 5 and 25 and was also sampled too. The two models were trained for 10 epochs on the meta-train set. For meta-testing we used two different sets: in the first one, we created 10^4 functions with the same parameters as the meta-train functions, and in the second we created 10^4 functions with frequency $\rho = 1.5$. We sampled 20 points from each meta-test function in the same x interval as contexts, and 1000 points as targets. We also evaluated the prediction error on the meta-train set in the same way. The models performed well on the meta-train set, as expected, and meta-generalized well to the test functions. However, they failed to meta-generalize to the test functions with the slightly different frequency F.1.

Table F.1: Mean squared error (MSE) of two meta-learning models on sinusoids. Values show mean and standard deviation.

	Train functions ($\rho = 1.0$)	Test functions ($\rho = 1.0$)	Test functions ($\rho = 1.5$)
MAML	0.39 (0.10)	0.394 (0.10)	3.53 (0.28)
CNP	0.01 (0.00)	0.005 (0.00)	6.69 (0.17)

G Random sampling of observations protects NPs from overfitting

Increasing the percentage of points sampled as contexts or targets leads to less unique function views and to more effective epochs. This causes overfitting, both in terms of R^2 (Figure G.1) and NLPD (see Figure 9).

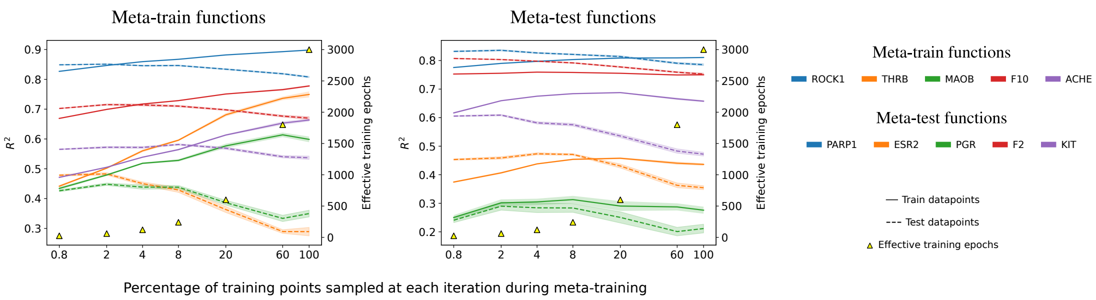

Fig. G.1: Increasing the percentage of points sampled as contexts or targets at each iteration leads to memorization of the labels from the train points of the train functions (left, solid lines) and a degradation of performance on the test points of the train functions (left, dashed line) and all points of the test functions (right, solid and dashed lines).

As the percentage of points sampled as contexts or targets increased, the calibration of the uncertainty estimates deteriorated substantially (Figure G.2).

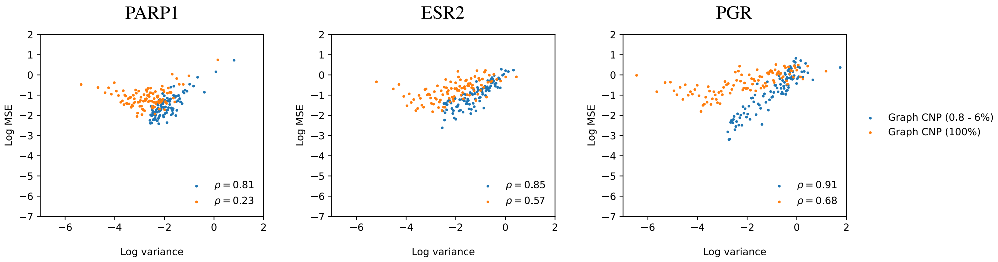

Fig. G.2: A MG-CNP meta-trained using a small fraction of the training points as contexts or targets at each iteration (blue) maintains a good calibration, as shown by the high Pearson correlation between the predicted log variance and the prediction error. In contrast, a MG-CNP meta-trained using all training points as context or targets (orange) displays poor calibration.

H Bayesian optimization objectives

Our BO experiments (Section 4.5) used the objective functions druglike F2 and selective JAK2 from the DOCKSTRING optimization benchmark [10].

- *Druglike F2* is a comparatively easy task that requires docking well to a single target and satisfying orally-bioavailable druglike properties according to QED:

  $$f_{\mathrm{F2}}(\ell) = s(\ell, \mathrm{F2}) + 10\big(1 - \mathrm{QED}(\ell)\big),$$

  where ℓ is a ligand molecule, and $s(\ell, p)$ is the docking score of ligand ℓ against protein p.

- *Selective JAK2* is a comparatively difficult taks that requires docking well against JAK2 and not against LCK. This is a hard task since the docking scores of these two kinases are very highly correlated. This objective reflects a real interest in the drug discovery community to design selective kinase inhibitors.

  $$f_{\mathrm{JAK2}}(\ell) = s(\ell, \mathrm{JAK2}) - \min\big(s(\ell, \mathrm{LCK}) - 8.1,\, 0\big) + 10\big(1 - \mathrm{QED}(\ell)\big)$$

  (Note that -8.1 is the median of LCK docking scores.)

References

[1] Garnelo, M., Rosenbaum, D., Maddison, C., Ramalho, T., Saxton, D., Shanahan, M., Teh, Y.W., Rezende, D., Eslami, S.A.: Conditional neural processes. In: International Conference on Machine Learning, pp. 1704–1713 (2018). PMLR

[2] Garnelo, M., Schwarz, J., Rosenbaum, D., Viola, F., Rezende, D.J., Eslami, S.M.A., Teh, Y.W.: Neural Processes. In: ICML 2018 Workshop on Theoretical Foundations and Applications of Deep Generative Models (2018)

[3] Mendez, D., Gaulton, A., Bento, A.P., Chambers, J., De Veij, M., Félix, E., Magariños, M.P., Mosquera, J.F., Mutowo, P., Nowotka, M., Gordillo-Marañón, M., Hunter, F., Junco, L., Mugumbate, G., Rodriguez-Lopez, M., Atkinson, F., Bosc, N., Radoux, C.J., Segura-Cabrera, A., Hersey, A., Leach, A.R.: ChEMBL: towards direct deposition of bioassay data. Nucleic Acids Res. **47**(D1), 930–940 (2019)

[4] Finn, C., Abbeel, P., Levine, S.: Model-Agnostic Meta-Learning for Fast Adaptation of Deep Networks. In: International Conference on Machine Learning, pp. 1126–1135 (2017). PMLR

[5] Raghu, A., Raghu, M., Bengio, S., Vinyals, O.: Rapid learning or feature reuse? towards understanding the effectiveness of MAML. In: International Conference on Learning Representations (2020)

[6] Antoniou, A., Edwards, H., Storkey, A.: How to train your MAML. In: International Conference on Learning Representations (2019)

[7] Paszke, A., Gross, S., Massa, F., Lerer, A., Bradbury, J., Chanan, G., Killeen, T., Lin, Z., Gimelshein, N., Antiga, L., Desmaison, A., Kopf, A., Yang, E., DeVito, Z., Raison, M., Tejani, A., Chilamkurthy, S., Steiner, B., Fang, L., Bai, J., Chintala, S.: Pytorch: An imperative style, high-performance deep learning library. In: Advances in Neural Information Processing Systems, vol. 32 (2019)

[8] Buitinck, L., Louppe, G., Blondel, M., Pedregosa, F., Mueller, A., Grisel, O., Niculae, V., Prettenhofer, P., Gramfort, A., Grobler, J., Layton, R., VanderPlas, J., Joly, A., Holt, B., Varoquaux, G.: API design for machine learning software: experiences from the scikit-learn project. In: ECML PKDD Workshop: Languages for Data Mining and Machine Learning, pp. 108–122 (2013)

[9] Gardner, J., Pleiss, G., Weinberger, K.Q., Bindel, D., Wilson, A.G.: Gpytorch: Blackbox matrix-matrix gaussian process inference with gpu acceleration. In: Advances in Neural Information Processing Systems, vol. 31 (2018)

[10] García-Ortegón, M., Simm, G.N.C., Tripp, A.J., Hernández-Lobato, J.M., Bender, A., Bacallado, S.: DOCKSTRING: Easy Molecular Docking Yields Better Benchmarks for Ligand Design. J. Chem. Inf. Model. **62**(15), 3486–3502 (2022)
